# Genome-Wide Association Study of Meiotic Recombination Phenotypes

**DOI:** 10.1534/g3.116.035766

**Published:** 2016-10-12

**Authors:** Ferdouse Begum, Reshmi Chowdhury, Vivian G. Cheung, Stephanie L. Sherman, Eleanor Feingold

**Affiliations:** * Department of Epidemiology, Johns Hopkins Bloomberg School of Public Health, Baltimore, Maryland 21205; † Department of Medicine, Johns Hopkins University School of Medicine, Baltimore, Maryland 21205; ‡ Department of Neurology, University of California, Los Angeles, California 90095; § Department of Human Genetics, University of Michigan, Ann Arbor, Michigan 48109; ** Department of Human Genetics, Emory University School of Medicine, Atlanta, Georgia 30322; †† Department of Human Genetics, University of Pittsburgh Graduate School of Public Health, Pittsburgh, Pennsylvania 15261; ‡‡ Department of Biostatistics, University of Pittsburgh Graduate School of Public Health, Pittsburgh, Pennsylvania 15261

**Keywords:** crossover, nondisjunction, GWAS meta-analysis, hotspot/nonhotspot, average recombination

## Abstract

Meiotic recombination is an essential step in gametogenesis, and is one that also generates genetic diversity. Genome-wide association studies (GWAS) and molecular studies have identified genes that influence of human meiotic recombination. *RNF212* is associated with total or average number of recombination events, and *PRDM9* is associated with the locations of hotspots, or sequences where crossing over appears to cluster. In addition, a common inversion on chromosome 17 is strongly associated with recombination. Other genes have been identified by GWAS, but those results have not been replicated. In this study, using new datasets, we characterized additional recombination phenotypes to uncover novel candidates and further dissect the role of already known loci. We used three datasets totaling 1562 two-generation families, including 3108 parents with 4304 children. We estimated five different recombination phenotypes including two novel phenotypes (average recombination counts within recombination hotspots and outside of hotspots) using dense SNP array genotype data. We then performed gender-specific and combined-sex genome-wide association studies (GWAS) meta-analyses. We replicated associations for several previously reported recombination genes, including *RNF212* and *PRDM9*. By looking specifically at recombination events outside of hotspots, we showed for the first time that *PRDM9* has different effects in males and females. We identified several new candidate loci, particularly for recombination events outside of hotspots. These include regions near the genes *SPINK6*, *EVC2*, *ARHGAP25*, and *DLGAP2*. This study expands our understanding of human meiotic recombination by characterizing additional features that vary across individuals, and identifying regulatory variants influencing the numbers and locations of recombination events.

Meiotic recombination is critical to successful human reproduction as it plays an essential role in the formation of gametes. It is also an important mechanism for ensuring genetic diversity at the population level. Unlike somatic recombination, meiotic recombination involves homologous DNA sequences. Meiotic recombination initiates with double-strand breaks of DNA, and repairs on the homologous DNA sequence of the homologous chromosome (Baudat *et al.* 2010). Too little recombination, absence of recombination, and recombination in certain high-risk locations, are all associated with aberrant meiotic outcomes, including chromosomal aneuploidies (Hou *et al.* 2013; Brieno-Enriquez and Cohen 2015; MacLennan *et al.* 2015). Chromosomal aneuploidies include trisomy and monosomy, which can result in pregnancy loss, intellectual disability, and birth defects.

Although the recombination process is tightly regulated, there is still considerable variation among individuals. There is gender-specific variability and individual-level variability at every scale (Broman *et al.* 1998; Cheung *et al.* 2007; Fledel-Alon *et al.* 2011). Recently, several studies have started to uncover the genetic determinants of variation in meiotic recombination in humans using either direct observation in gametes or inferring recombination based on family genotype data (Chowdhury *et al.* 2009; Kong *et al.* 2010, 2014; Fledel-Alon *et al.* 2011) or population-level data (Myers *et al.* 2005). Different studies have focused on different aspects of trait variation, such as average number of recombination events, location and frequency of the recombination in different areas on the genome, and different patterns in males and females, etc.

The most commonly studied recombination phenotype is average recombination count (ARC) over multiple gametes in a single proband (parent). One gene, *RNF212*, has been conclusively shown to affect overall recombination in ARC (Coop *et al.* 2008; Kong *et al.* 2008, 2014; Chowdhury *et al.* 2009; Fledel-Alon *et al.* 2011). Kong *et al.* (2008) first reported the *RNF212* gene in a GWAS study conducted in an Icelandic population, and showed that specific SNPs in *RNF212* have opposite effects on male and female recombination rates. This result was later replicated by other studies (Coop *et al.* 2008; Kong *et al.* 2008, 2014; Chowdhury *et al.* 2009; Fledel-Alon *et al.* 2011). In addition to specific genes, an inversion on chromosome 17q21.31 is also associated with female recombination rate (Kong *et al.* 2008, 2014; Chowdhury *et al.* 2009; Fledel-Alon *et al.* 2011). Other genes putatively associated with ARC include *KIAA1462* in females, and *UGCG* and *NUB1* in males (Chowdhury *et al.* 2009; Fledel-Alon *et al.* 2011), but these have failed to replicate in other studies (Fledel-Alon *et al.* 2011; Kong *et al.* 2014). Most recently, Kong *et al.* (2014) reported eight new variants (including two rare variants) associated with recombination in the Icelandic population. This latter study used methods based on long-range haplotyping uniquely applicable to the Icelandic population. Because of the extremely large sample size, and the highly significant p values reported by Kong *et al.* (2014), it is likely that most or all of these are true positive associations, at least in this population, but they have not yet been examined in any other population (Kong *et al.* 2014).

Several studies have shown that, in addition to the total recombination rate, the location of recombination events is also under genetic control. Abnormal recombination location has been associated with improper chromosomal segregation (Kimura *et al.* 2006; Cheung *et al.* 2010). Based on historical population-based information as represented in patterns of linkage disequilibrium (LD), the frequency of recombination events is higher at some locations of the genome. These 1–2 kb areas of the genome are known as “hotspots” (Kauppiz *et al.* 2004; Neale 2010). Hotspot areas may be determined by multiple factors such as presence of a particular motif in the hotspot regions, presence of epigenetic factors, and *trans*-acting loci (Sandovici and Sapienza 2010).

*PRDM9* has been shown in several recent studies to affect recombination within hotspots. Activity of various alleles of *PRDM9* differs, thus genotype may affect genome-wide hotspot activity (Berg *et al.* 2010; 2011; Kong *et al.* 2010; Hinch *et al.* 2011; Segurel *et al.* 2011). The role of *PRDM9* is not limited to human recombination hotspot usage. A recent study showed that *PRDM9* is also involved with nonexchange gene conversion (Sarbajna *et al.* 2012). All of these findings suggest there are other unknown determinants that will add to our understanding of the mechanism of *PRDM9* and its role in human recombination and hotspot usage.

The human consensus PRDM9 allele is predicted to recognize the 13-mer motif enriched at human hotspots, and considered as one of the major regulators of meiotic recombination hotspots (Yang *et al.* 2014); thus, the percent of recombination near these motifs may show individual variability that is genetically determined (although the motif issue is itself controversial) (Kong *et al.* 2014). From the hotspot locations, initially a list of motifs including 9-mer and 7-mer later extended to degenerate 13-mer motifs containing zinc finger-binding arrays has been discovered (Myers *et al.* 2008; Yang *et al.* 2014).

The goals of our study are to find additional recombination genes, and to gain greater understanding of previously discovered genes. In particular, we consider new phenotypes related to hotspot usage to dissect further the genetic architecture of recombination control. We consider percent of recombination occurring in historical hotspots (HS_PCT), average count of recombination occurring in historical hotspots (HS_CNT), average count of recombination occurring outside of historical hotspots (NHS_CNT), and percentage of recombination occurring near the putative motif (MOTIF). The rationale for looking separately at recombination in and out of hotspots, and looking at hotspot recombination as both a percentage and a count, is that these different measures may add insight about the effects of genes. For example, if a variant increases recombination in hotspots but decreases recombination outside of hotspots, there may be a compensatory regulatory mechanism acting to keep total recombination constant. We studied all phenotypes separately in males and females, and also performed combined-sex analyses. Most previous studies of the ARC phenotype have found very different effects in males and females, while previous studies of hotspot phenotypes have shown similar effects in both sexes (Berg *et al.* 2010; Kong *et al.* 2014). In addition, we focus on the question of whether the genes discovered by Kong *et al.* (2014) are associated with recombination phenotypes in a European descent population, given that some of them are relatively rare variants in the Icelandic population.

## Materials and Methods

### Study population and samples

This study included three populations: the Geneva Dental Caries Study (GDCS) (Shaffer *et al.* 2011), the Autism Genetic Resource Exchange (AGRE) (Weiss 2008), and the Framingham Heart Study (FHS) (Dawber *et al.* 1951). The GDCS and FHS samples were ascertained without regard to any particular phenotype. There is no known relationship between autism and meiotic recombination. The GDCS and AGRE samples were genotyped on the Illumina Human610-Quad Beadchip, and FHS samples were genotyped on the Affymetrix 5.0 chip. After quality control, final analysis was limited to autosomes only, and a total of 551,227 SNPs, 520,018 SNPs, and 388,060 SNPs from GDCS, AGRE, and FHS datasets, respectively.

### Pedigrees

Two-generation nuclear pedigrees with two or more children were used for this study; 171 families came from GDCS, 737 from AGRE, and 654 from FHS. Genotype data on each family were used to score recombination in each parent. Quantitative measures of meiotic recombination in the parents were then used for the GWAS analyses.

### Phenotypes

Recombination events in each parent of a nuclear family were called according to the method described in Chowdhury *et al.* (2009). Briefly, the method is as follows: first, the set of informative markers is identified in each family. A locus is informative if one parent is homozygous and another is heterozygous. Among two or more children, one is considered as the reference child, and, in a sibling pair, a switch from one allele to another allele in a particular parental haplotype along the chromosome will indicate a recombination in the heterozygous parent. A recombination thus observed in a sibling pair cannot be assigned to a specific offspring, but we do not need to do so since we are calculating the recombination phenotype for the parent. When there are three or more siblings, recombinations observed in more than one pair can be resolved as described by Chowdhury *et al.* (2009) to correctly score recombination in the parent. To avoid spurious double-recombinants due to genotyping error, we required five or more consecutive markers to call each observed recombination event.

From the recombination data, we calculated five different recombination phenotypes: ARC, HS_PCT, HS_CNT, NHS_CNT, and MOTIF. A set of predefined historic hotspot regions identified by HapMap project (International HapMap Consortium 2007) was used to calculate the three phenotypes related to hotspots: HS_PCT, HS_CNT, and NHS_CNT. We limit our hotspot phenotype analysis only to the recombination loci with 30 kb resolution. Precise definitions of these phenotypes are as follows:

  1.ARC=(total recombination in all children of the parent/number of children)
  $$\begin{array}{c} 2.\mathrm{HS}\text{\_}\mathrm{PCT}=(\text{total number of recombination overlapping recognized hotspots in all}\\ \text{children of the parent})/\left(\text{total recombination in all children of the parent}\right) \end{array}$$
  $$\begin{array}{c} 3.\mathrm{HS}\text{\_}\mathrm{CNT}=(\text{total number of recombination overlapping hotspots in all}\\ \text{children of the parent})/\left(\text{number of children}\right) \end{array}$$
  $$\begin{array}{c} 4.\mathrm{NHS}\text{\_}\mathrm{CNT}=(\text{total number of recombination overlapping non-hotspots in all}\\ \text{children of the parent})/\left(\text{number of children}\right) \end{array}$$
  $$\begin{array}{c} 5.\mathrm{MOTIF}=(\text{total number of recombination with motif in all children of the}\\ \text{parent})/\left(\text{total recombination events in all children}\right) \end{array}$$

### Genotypes, error checking, and data handling

For the GDCS dataset, 589,735 SNPs were released by the Center for Inherited Disease Research (CIDR). The AGRE dataset had 520,018 SNPs, and FHS had 388,060 SNPs available for analysis. To ensure the quality, an extensive data cleaning was performed for these datasets. Full details of data cleaning steps for GDCS can be found in Geneva consortium website (https://www.genome.gov/27550876/). Detailed data cleaning steps for AGRE and FHS datasets are presented in Chowdhury *et al.* (2009). Briefly, measures of identity-by-descent were used to verify relationships, SNP intensities of X- and Y-chromosomes were used to verify gender, and principal component analysis (PCA) was used to summarize genetic ancestry. Two thresholds used in the analysis are a Hardy-Weinberg disequilibrium cut-off of p < 0.0001, and minimum minor allele frequency cut-off of <2% for all SNPs.

### Genome-wide association studies

To identify genes or SNPs associated with different aspects of recombination, we conducted three genome-wide association studies for each phenotype; we conducted separate male and female analyses, as well as performing a combined analysis. We used PLINK (http://pngu.mgh.harvard.edu/∼purcell/plink/) to conduct all GWAS using an additive genetic model. All of our phenotypes are continuous; so we used the linear regression option in PLINK for the association tests. As per significance level of association studies, we used the threshold with p < 10^−07^ as genome-wide significant.

We combined the AGRE and GDCS GWAS results using meta-analysis instead of combining all three datasets, because the AGRE and GDCS datasets were genotyped on the same platform (Illumina 610 chip), while the FHS dataset was genotyped on the Affymetrix 5.0 chip, which has a very different coverage profile. Because the Affymetrix 5.0 platform has very different coverage than the Illumina platform in a number of key regions, we did not impute genotypes, since imputation does not “fix” lack of coverage (Begum *et al.* 2012). We used fixed effects meta-analysis to combine the GDCS and AGRE datasets, which has been shown to perform very similarly to mega-analysis (directly combining datasets), but is slightly more robust to population differences in the phenotype (Lin and Zeng 2010; Sung *et al.* 2014). We performed GWAS meta-analysis for each gender separately, and also performed combined-sex GWAS meta-analysis using the software METAL (Willer *et al.* 2010). We used *R* for most of the data analysis, and LocusZoom (Pruim *et al.* 2010) to plot the data for each genomic region. We then used the FHS dataset for qualitative replication in regions suggestive or significant in the meta-analyses.

### Data availability

We used publicly available genotyping datasets for this study. GDCS, AGRE, and FHS data supporting these findings are available through dbGaP repository (phs000095.v1.p1 [https://www.ncbi.nlm.nih.gov/projects/gap/cgi-bin/study.cgi?study_id=phs000095.v1.p1], phs000267.v1.p1 [https://www.ncbi.nlm.nih.gov/projects/gap/cgi-bin/study.cgi?study_id=phs000267.v1.p1], and phs000342.v16.p10 [https://www.ncbi.nlm.nih.gov/projects/gap/cgi-bin/study.cgi?study_id=phs000342.v16.p10], respectively).

## Results

Important characteristics of these datasets are summarized in Table 1. The GDCS dataset has not been used previously in any published study of recombination. The AGRE resource was used in Chowdhury *et al.* (2009), but the dataset used here is larger, and was genotyped with a denser GWAS array chip. The FHS dataset used here is the same as that used in Chowdhury *et al.* (2009).

**Table 1:** Dataset information

Dataset	GDCS	AGRE	FHS
Sample size	342	1473	1293
Male	171	736	639
Female	171	737	654
Chip type	Illumina Human660-Quad Beadchip	Illumina Human660-Quad Beadchip	5.0 Affy chip
Total SNPs	551,227	520,018	388,060
Mean # of children	2.46	2.70	2.89
ARC			
Male	27.38	26.43	27.72
Female	44.05	40.69	42.98
Recombination ratio (Female: Male)	1.61	1.54	1.52
Hotspot usage (average percent)			
Male	39%	50%	32%
Female	36%	42%	30%
HS_CNT (limited to 30-kb intervals)			
Male	9.03	11.80	4.73
Female	13.66	17.36	7.06
NHS_CNT (limited to 30-kb intervals)			
Male	14.40	11.69	10.47
Female	24.81	18.83	16.77
Motif overlap (average percent)			
Male	46%	46%	47%
Female	42%	45%	47%

In GDCS, a total of 421 children were used to score recombination for 171 male and 171 female meioses. Similarly, 1987 and 1858 children were used to score recombination in 736 male and 737 female meioses in AGRE, and 639 male and 654 female meioses in FHS. We used nuclear families with two or more children to score recombination for each of the parents. p values from GCDS and AGRE were combined by meta-analysis for each sex individually, and for both sexes combined. FHS was then used as a replication dataset at the gene level.

### GWAS for new recombination phenotypes

For each of the recombination phenotypes, we performed a GWAS in males (meta-analysis of AGRE and GCDS), a GWAS in females (similarly), and a GWAS combining both datasets for both sexes. The 579,043 SNPs overlapping between GDCS and AGRE datasets are included in this meta-analysis. The most significant new results for each phenotype are presented below. We used two different cut-offs for statistical significance in our GWAS analyses: genome-wide significant with p < 10^−07^, and p value between 10^−05^ < p < 10^−07^ as a suggestive signal. Following the new results, the subsequent section discusses replication of previously reported associations. In discussing replication of previously published results, we considered significance levels appropriate for candidate gene analyses. This is followed by a qualitative description of replication in the FHS dataset. The final section of results examines our associations across all five phenotypes in order to infer new information about *RNF212* and *PRDM9*.

#### Average recombination count:

**Figure 1:**
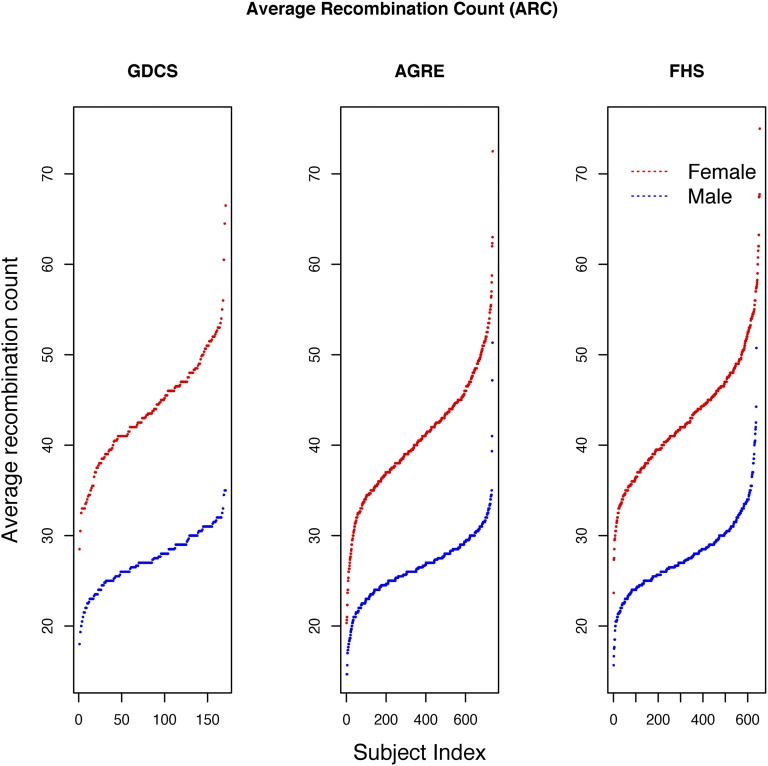
Distribution of ARC phenotype in three datasets: distributions of ARCs for three datasets are presented in three panels. Red dots, female ARC; blue dots, male ARC.

**Figure 2:**
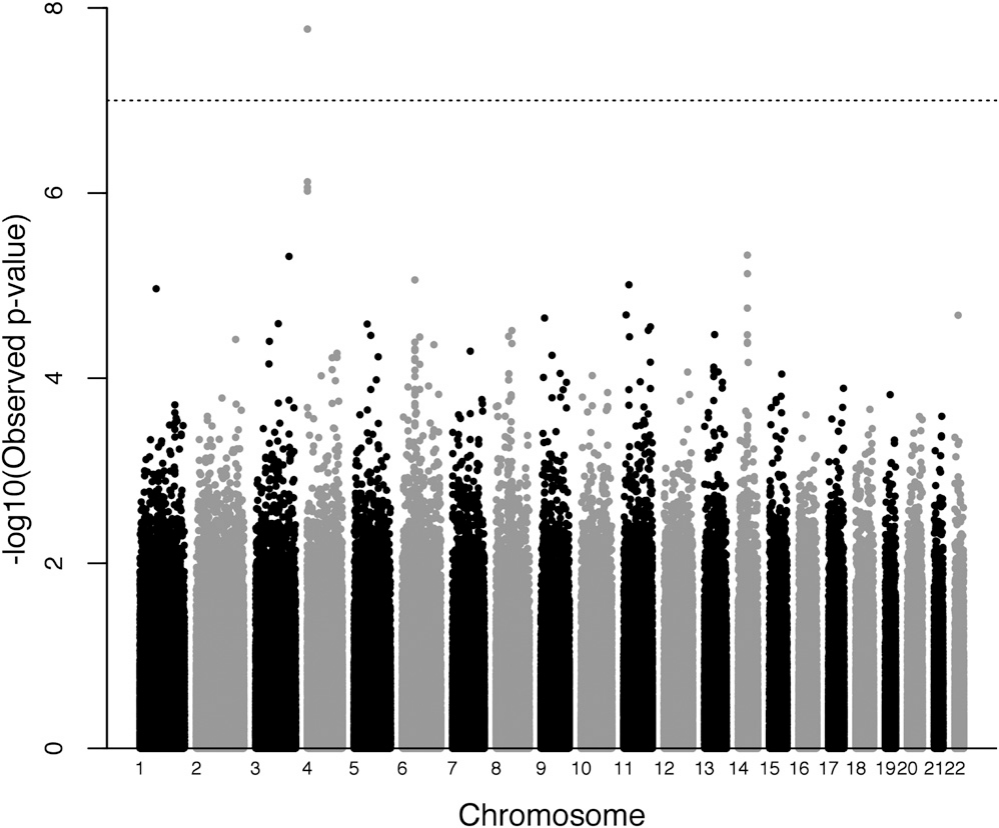
Manhattan plot of genome-wide association scan for phenotype ARC (male only analysis): each point represents a SNP. The black dotted line represents the genome-wide significance level for Bonferroni correction; 22 autosomes are represented with black and gray shades for visual clarity.

**Figure 3:**
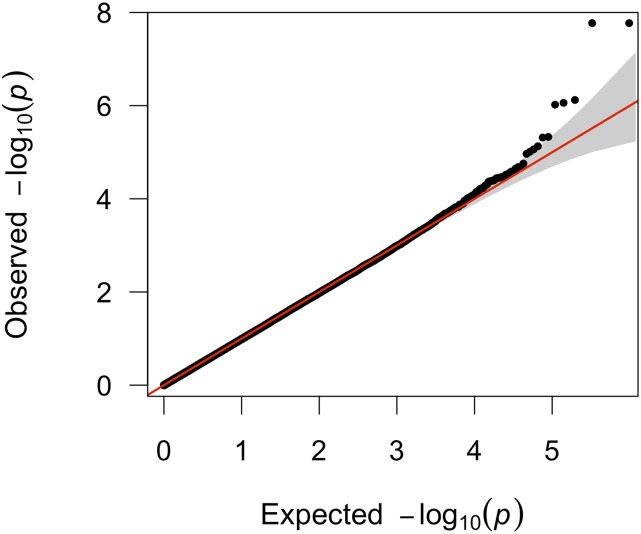
Quantile-Quantile (Q-Q) plot of the genome-wide association of phenotype ARC (male only analysis): −log_10_ transformed observed p values (*y*-axis) were plotted against −log_10_ transformed expected p values (*x*-axis).

ARCs for three different datasets are presented in Table 1. The ARCs for each of these studies, and the variation between males and females, are quite consistent with previous studies of human meiotic recombination (Chowdhury *et al.* 2009; Kong *et al.* 2010, 2014; Fledel-Alon *et al.* 2011). The distribution of the male and female average recombination counts per meiosis is presented in Figure 1. The top five most highly associated SNPs for all GWAS analyses of the ARC phenotype (male, female, and combined-sex) are listed in Table 2, which also includes nearby flanking genes for each region. In the male analysis, *RNF212* was the most significant gene (p = 1.695e^−08^). Males and females have estimated effects in opposite directions, which is consistent with the previous literature. The Manhattan plot for the male-only analysis is presented in Figure 2, and the QQ plot of the same analysis is presented in Figure 3. Manhattan and QQ plots of the female meta-analysis, and the pooled meta-analysis results are presented in Supplemental Material, Figure S1, Figure S2, Figure S3, and Figure S4.

**Table 2:** SNPs with lowest p values for ARC

Analysis Type	SNP	Chr	BP	p Value	Direction	Gene List
Combined	*rs4974601*	4	1,085,409	2.76E−07	− − + −	*RNF212*
	*rs444996*	8	40,298,364	3.34E−06	++++	*C8orf4, ZMAT4*
	*rs724055*	22	29,005,922	3.60E−06	− +++	*LIF, OSM, GATSL3, TBC1D10A, SF3A1, CCDC157, SEC14L2, MTP18, HORMAD2*
	*rs1996483*	3	167,607,627	6.31E−06	− +++	*chr3: 167107628−168107628*
	*rs9381359*	6	45,098,602	7.28E−06	++++	*SUPT3H, MIR586*
Female	*rs497793*	3	154,948,531	3.47E−07	++	*C3orf79, SGEF*
	*rs12903708*	15	58,380,596	1.13E−06	++	*FOXB1, ANXA2, NARG2*
	*rs2974754*	19	12,922,982	2.43E−06	++	*FARSA, DAND5, CALR, RAD23A*
	*rs4879584*	9	32,402,621	3.26E−06	++	*ACO1, DDX58*
	*rs9572559*	13	70,310,774	3.79E−06	− −	*chr13: 69810775−70810775*
Male	***rs4974601***	**4**	**1,085,409**	**1.695e−08**	**− −**	***RNF212***
	*rs1951371*	14	59,425,467	4.69E−06	− −	*RTN1*
	*rs1996483*	3	167,607,627	4.84E−06	++	*chr3: 167107628−168107628*
	*rs1418433*	6	44,860,545	8.68E−06	++	*SUPT3H, SPATS1, AARS2*
	*rs1035699*	11	19,713,338	9.80E−06	− −	*NAV2, LOC100126784*

Column 6 of the table represents the direction of the effect size of each SNP presented in column 2 in each study. In combined analysis, studies were included in the following order (GDCS female, GDCS male, AGRE female, and AGRE male). In female only analysis, first position in the direction column is for GDCS female, and the 2nd position is for AGRE female; the same ordering is used in male only analysis, and for rest of the phenotypes.

#### Percent of recombination occurring in hotspots:

**Figure 4:**
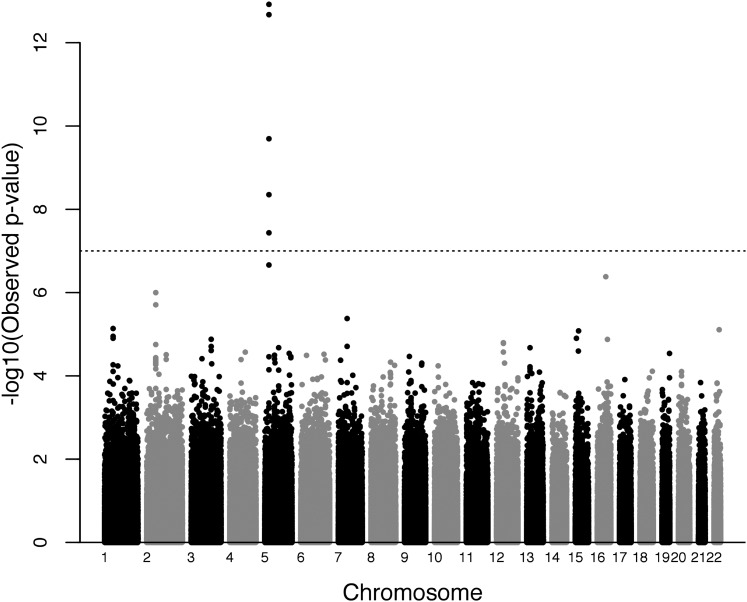
Manhattan plot of genome-wide association scan for phenotype HS_PCT (male and female combined analysis): each point represents a SNP. The black dotted line represents the genome-wide significance level for Bonferroni correction; 22 autosomes are represented with black and gray shades for visual clarity.

**Figure 5:**
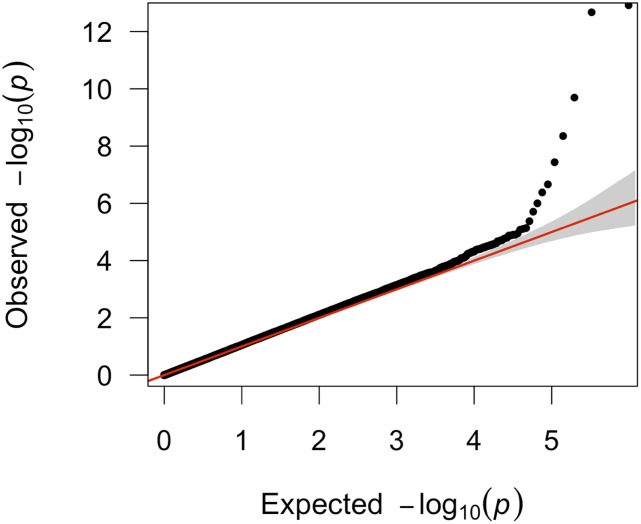
Quantile-Quantile (Q-Q) plot of the genome-wide association of phenotype HS_PCT (male and female combined analysis): −log_10_ transformed observed p values (*y*-axis) were plotted against −log_10_ transformed expected p values (*x*-axis).

Distribution of the HS_PCT phenotype is presented in Figure S5. For the HS_PCT phenotype, the top signals for male only, female only, and combined-sex GWAS analysis are presented in Table 3. The strongest association (p = 1.20e−13) was with multiple SNPs in and near the *PRDM9* gene in the combined-sex analysis (top SNP reported). In the separate male and female analyses, *PRDM9* was also among the most statistically significant results. Manhattan plots and QQ plots for female and male are presented in Figure S6, Figure S7, Figure S8, and Figure S9. Figure 4 presents the Manhattan plot of the combined-sex analysis, with the QQ plot in Figure 5. It is notable that other regions showed similar levels of association as observed for *PRDM9*, particularly in males.

**Table 3:** SNPs with lowest p values for HS_PCT

Analysis Type	SNP	Chr	BP	p Value	Direction	Gene List
Combined	***rs1603084***	**5**	**23,567,950**	**1.20E−13**	**− − − −**	***PRDM9***
	*rs12445855*	16	68,068,843	4.16E−07	− − − −	*CYB5B, MIR1538, TERF2, NFAT5*
	*rs972847*	2	50,227,778	1.00E−06	++++	*NRXN1*
	*rs13232367*	7	43,342,734	4.20E−06	++++	*HECW1*
	*rs2716140*	1	59,244,984	7.29E−06	++++	*LOC729467, JUN*
Female	***rs1603084***	**5**	**23,567,950**	**2.54E−09**	**− −**	***PRDM9***
	*rs12445855*	16	68,068,843	4.64E−07	− −	*CYB5B, MIR1538, TERF2, NFAT5*
	*rs949029*	18	50,885,623	1.32E−05	++	*CCDC68, RAB27B, TCF4*
	*rs355926*	16	65,270,601	1.36E−05	− −	*CMTM4, DYNC1LI2, CCDC79*
	*rs2292305*	15	37,668,113	1.58E−05	− −	*THBS1, FSIP1*
Male	*rs10996809*	10	67,413,658	2.91E−06	++	*CTNNA3*
	*rs12958111*	18	71,979,757	6.88E−06	++	*ZNF516*
	*rs1874165*	5	23,559,104	7.59E−06	− −	*PRDM9*
	*rs13378443*	13	92,254,975	8.22E−06	++	*GPC5, GPC6*
	*rs1603084*	5	23,567,950	9.79E−06	− −	*PRDM9*

Column 6 of the table represents the direction of the effect size of each SNP presented in column 2 in each study. In combined analysis, studies were included in the following order (GDCS female, GDCS male, AGRE female, and AGRE male). In female only analysis, first position in the direction column is for GDCS female and the 2nd position is for AGRE female and same ordering is used in male only analysis and for the remaining phenotypes.

#### Average count of recombinants in hotspots:

Our third phenotype was HS_CNT. Distribution of the HS_CNT phenotype is presented in Figure S10. Table 4 shows the top five hits for single-sex and combined-sex GWAS meta-analyses. Males showed a stronger effect of *PRDM9* on HS_CNT than did females, the opposite of what was observed for HS_PCT. Other suggestive SNPs for HS_CNT had very minimal overlap with the suggested SNPs for HS_PCT. Among the top hits for the male-only analysis of HS_CNT was *RNF212*, while the top hit in the combined analysis was in *PRDM9*. The top hit for the female-only analysis was in an intergenic region. Manhattan plots and QQ plots for HS_CNT are presented in Figure S11, Figure S12, Figure S13, Figure S14, Figure S15, and Figure S16.

**Table 4:** SNPs with lowest p values for HS_CNT

Analysis Type	SNP	Chr	BP	p Value	Direction	Gene list
Combined	***rs1874165***	**5**	**23,559,104**	**3.80E−08**	**− − − −**	***PRDM9***
	*rs2764928*	1	59,195,376	3.69E−07	++++	*JUN, LOC729467*
	*rs7650855*	3	73,602,421	1.47E−06	++++	*PDZRN3*
	*rs16863103*	2	15,918,176	1.77E−06	++++	*DDX1, MYCNOS, MYCN*
	*rs13253524*	8	119,294,947	1.88E−06	− − − −	*EXT1, SAMD12*
Female	*rs1242541*	14	82,275,789	2.87E−06	++	*chr14: 81775790−82775790*
	*rs2959776*	8	6,415,275	5.40E−06	++	*MCPH1, ANGPT2, AGPAT5*
	*rs2569491*	19	56,276,727	8.57E−06	++	*KLK12, KLK13, KLK14, CTU1, SIGLEC9, SIGLEC7, SIGLECP3*
	*rs6720182*	2	68,848,001	1.24E−05	− −	*PROKR1, ARHGAP25, BMP10*
	*rs4797343*	18	8,964,854	1.47E−05	++	*KIAA0802, NDUFV2*
Male	*rs10958702*	8	41,865,459	2.06E−06	− −	*ANK1*
	*rs13378443*	13	92,254,975	3.51E−06	++	*GPC5, GPC6*
	*rs169266*	1	167,090,734	4.47E−06	++	*DPT, MGC4473, ATP1B1*
	*rs1874165*	5	23,559,104	4.62E−06	− −	*PRDM9*
	*rs325702*	11	6,216,076	4.87E−06	++	*OR56B4, OR52B2, OR52W1, FAM160A2, PRKCDBP*

Column 6 of the table represents the direction of the effect size of each SNP presented in column 2 in each study. In combined analysis, studies were included in the following order (GDCS female, GDCS male, AGRE female, and AGRE male). In female only analysis, first position in the direction column is for GDCS female, and the 2nd position is for AGRE female and same ordering is used in male only analysis and for the remaining phenotypes.

#### Average count of recombinants in nonhotspot areas:

In the analysis of recombination events outside of hotspots, we looked at NHS_CNT. Distribution of the NHS_CNT phenotype is presented in Figure S17. The top five SNPs from each analysis are presented in Table 5. In the combined-sex analysis, one of the SNPs (chr5: *rs12186491*) was genome-wide significant (p = 6.36E−08), and this SNP is in the gene *SPINK6*, which is a serine protease inhibitor. The next most significant hit was in *PRDM9*. In female-only analysis, none of the SNPs were genome-wide significant. In male-only analysis, one SNP (chr4: *rs10937651*) in *EVC2* showed genome-wide significance. *EVC2* is a protein coding gene, and related to bone formation and skeletal development, and is well known as causal for Ellis-van Creveld syndrome, which has clinical features including limb and facial abnormalities, and heart defects (D’Asdia *et al.* 2013; Kamal *et al.* 2013). The Manhattan plots and QQ plots are presented in Figure S18, Figure S19, Figure S20, Figure S21, Figure S22, and Figure S23.

**Table 5:** SNPs with lowest p values for NHS_CNT

Analysis Type	SNP	Chr	BP	p Value	Direction	Gene List
Combined	***rs12186491***	**5**	**147,573,689**	**6.36E−08**	**++++**	***SPINK5L2, SPINK6, SPINK5L3, SPINK7, SPINK9***
	*rs2914263*	5	23,488,680	1.16E−07	++++	*PRDM9*
	*rs10937651*	4	5,596,712	1.65E−07	++++	*STK32B, C4orf6, EVC2*
	*rs7403622*	15	31,977,777	2.16E−07	++++	*AVEN, RYR3*
	*rs11966986*	6	56,628,268	3.19E−07	++++	*DST*
Female	*rs3129595*	13	21,458,281	2.28E−06	++	*FGF9*
	*rs7873463*	9	4,211,297	3.23E−06	++	*GLIS3*
	*rs2065079*	14	50,320,526	4.22E−06	++	*SAV1, NIN, ABHD12B, PYGL*
	*rs1861509*	2	205,885,994	4.65E−06	++	*PARD3B*
	*rs1571463*	20	54,859,767	5.88E−06	++	*TFAP2C, BMP7*
Male	***rs10937651***	**4**	**5,596,712**	**5.16E−08**	**++**	***STK32B, C4orf6, EVC2***
	*rs11966986*	6	56,628,268	7.41E−07	++	*DST*
	*rs6994475*	8	1,260,832	1.67E−06	++	*DLGAP2*
	*rs7900873*	10	14,903,869	2.30E−06	++	*CDNF, HSPA14, SUV39H2*
	*rs1795514*	12	79,856,997	2.56E−06	++	*LIN7A, MIR617, MIR618*

Column 6 of the table represents the direction of the effect size of each SNP presented in column 2 in each study. In combined analysis, studies were included in the following order (GDCS female, GDCS male, AGRE female, and AGRE male). In female only analysis, first position in the direction column is for GDCS female, and the 2nd position is for AGRE female and same ordering is used in male only analysis and for the remaining phenotypes.

#### Percent of recombination occurring near the motif:

The distribution of the MOTIF phenotype is presented in Figure S24. As our last phenotype, we looked at the percent of recombination occurring near the 13 bpr MOTIF. Table S1 lists top hits from each analysis (female-only, male-only, and combined-sex). The Manhattan plots and QQ plots are presented in Figure S25, Figure S26, Figure S27, and Figure S28, Figure S29, and Figure S30.

### Replication of previously reported genes

Over the past decade, several studies have characterized meiotic recombination variation, and identified a handful of genes/loci associated with different aspects of recombination. We replicated two most well known genes (*PRDM9* and *RNF212*).

In addition to *PRDM9* and *RNF212*, the most recent study by Kong *et al.* (2014) nominated eight new loci as being associated with total recombination, including some rare variants. While they also examined recombination events within hotspots, they found no new evidence of association with hotspot recombination. Because of the enormous sample size used (35,927 parents, and 71,929 offspring), most of these loci were highly significant, and are likely to be true associations with recombination in the Icelandic population. However, these have not been examined in other populations.

Table S2 qualitatively summarizes our results at the gene level for reported top hits from Kong *et al.* (2014). LocusZoom plots for selected loci are presented in Figure S31. Our sample size is much smaller that that of Kong *et al.* (2014), and our study population is from the United States (primarily of European ancestry), but we were able to see evidence of replication of several of their loci. Poor coverage limited our ability to replicate others. Though our analysis was limited to only common markers, when we looked at the gene level replication, we were able to replicate evidence for *CPLX1* (p ∼ 10^−07^) and *MSH4* (p ∼ 10^−03^), which carried rare variants in the data of Kong *et al.* (2014).

SNPs in the inverted segment on chromosome 17 showed consistent (lowest p ∼ 10^−4^) hits of replication across three phenotypes (ARC, HS_CNT, and MOTIF) in females, but not in males, which is consistent with Kong *et al.* (2010, 2014). Different SNPs in the region were associated with different phenotypes, however. Selected LocusZoom plots for that region across phenotypes are presented in Figure S32, and the plot for HS_CNT female is presented in Figure 8A.

Other previous GWAS studies of recombination have also reported several possible associations, including *NUB1*, *UGCG*, and SNP (chr5: *rs17542943*) for male average recombination counts (Chowdhury *et al.* 2009; Fledel-Alon *et al.* 2011). Similarly, previously reported genes for female average recombination include *PDZK1*, *KIAA1462*, *CRHR1*, *LRRC37A*, *OBSCN*, and SNP (chr9: *rs10985535*) (Chowdhury *et al.* 2009; Fledel-Alon *et al.* 2011). LocusZoom plots of these previously reported genes from our male and female analyses are presented in Figure S33 and Figure S34, respectively. In males, the *UGCG* gene replicated moderately (p = 1.34E−4), and others showed hints of replication. In females, only *CRHR1* (p ∼ 10^−4^) and *KIAA1462* (p ∼ 10^−3^) showed suggestive replication.

### Replication of GDCS and AGRE study findings in FHS study

To support our GWAS meta-analysis findings in GCDS and AGRE, we examined ∼150 regions of interest in the FHS dataset that included at least the top 10 significant SNPs from the fixed effect meta-analyses of GDCS and AGRE for each phenotype, and made LocusZoom plots in the FHS dataset, totaling around 150 LocusZoom plots. We compared male-only analysis with FHS male GWAS results, and female-only analysis with FHS female GWAS results. To compare combined-sex analysis, we combined FHS male and female analyses using fixed effect meta-analysis. Because the FHS dataset, and the two other datasets examined here, had limited SNP overlap, we performed this replication analysis at the gene level. We did not impute because imputation would not overcome the problem of significantly different coverage for the two chips. Since many of the SNPs/genes of our interest were not among the top hits of FHS dataset (for example, the top hits for the phenotype ARC in FHS dataset presented in Table S3), instead of presenting top hits for each phenotype for the FHS dataset, we extracted our SNPs/gene of interest from the FHS dataset and provide p values as well as LocusZoom plots.

**Figure 6:**
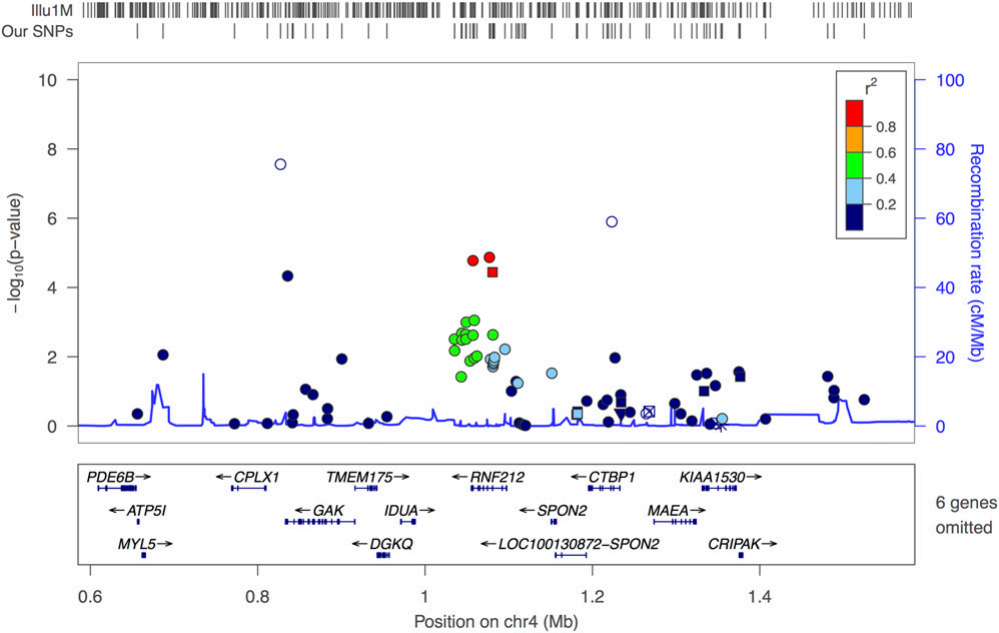
RNF212 (male) in FHS dataset: this figure displays 1000 kb regions around *RNF212* gene. In FHS dataset, the RNF212 gene is well covered. The SNPs are color-coded according to HapMap Phase II CEU LD pattern between SNPs presented in rectangular box in upper right corner. Known genes, and orientations are plotted below the SNPs. HapMap recombination rates are shown with a blue line behind the SNPs. SNP coverage in FHS datasets and Illumina 1 million chip is noted by tick marks above the plot.

For the ARC phenotype, the only replication observed in the FHS dataset was for *RNF212* in males (p ∼ 10^−5^; Figure 6). In males, a SNP near *NAV2* (5th significant SNP *rs1035699*, Table 2) also showed p ∼ 10^−5^. Only three SNPs of the 11 most significant in AGRE/GCDS were genotyped in the FHS dataset. Among the eight other SNPs, two were tagged by SNPs with strong LD (0.8 < *r*^2^ < 1.0) in FHS, and four were in medium to high LD.

**Figure 7:**
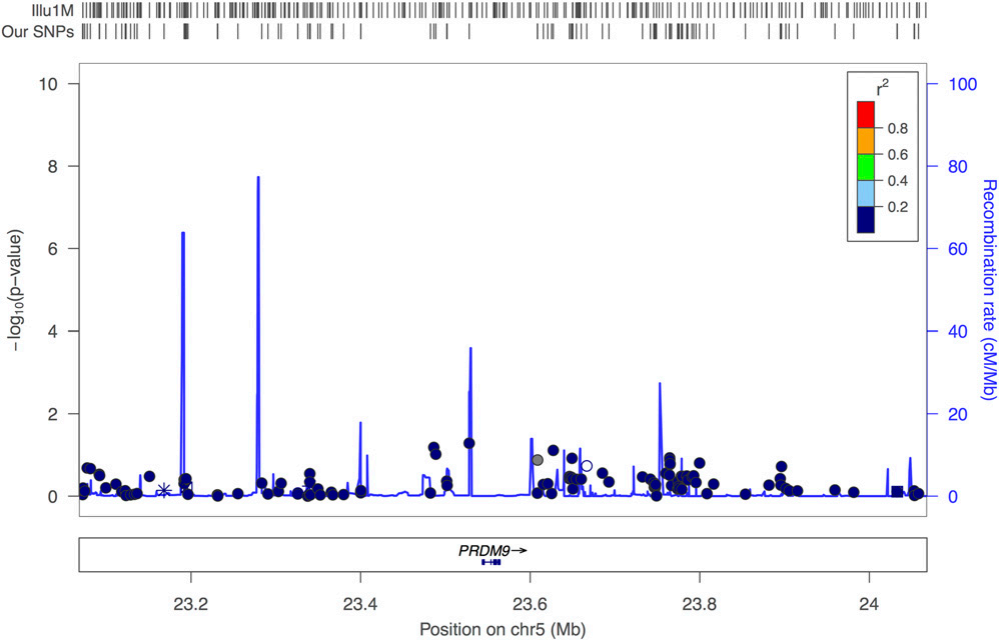
PRDM9 (male and female combined analysis) in FHS dataset: this figure displays 1000 kb regions around the *PRDM9* gene. In the FHS dataset, the *PRDM9* gene is not covered. The SNPs are color-coded according to HapMap Phase II CEU LD pattern between SNPs presented in rectangular box in upper right corner. Known genes, and orientations are plotted below the SNPs. HapMap recombination rates has been shown with a blue line behind the SNPs. SNP coverage in FHS datasets, and Illumina 1 million chip is noted by tick marks above the plot.

For our HS_PCT, HS_CNT and NHS_CNT phenotypes, the *PRDM9* gene was the center of interest. However the FHS dataset showed no SNP in *PRDM9* significantly associated with any of these phenotypes due to extremely poor coverage (see Figure 7).

For HS_CNT, a few of the top results from the AGRE/GCDS meta-analyses showed gene-level replication in FHS. For HS_CNT, in females, the fourth significant SNP was in *ARHGAP25*. In the FHS dataset, several SNPs on *ARHGAP25* showed p ∼ 10^−4^. And SNPs near *SULF2* (10th significant hit) showed p ∼ 10^−3^ in the FHS dataset. In males, the 2nd most significant hit was *rs13378443* (nearby genes *GPC5*, *GPC6*). In FHS dataset, SNPs near *GPC5* showed p ∼ 10^−3^.

For the NHS_CNT phenotype, there were again some gene-level replications in the FHS dataset, including *rs12186491* on *SPINK6* (p = 1.1e^−04^) is presented in Figure S35. In male analysis, the top significant hit in the GCDS/AGRE meta-analysis was *EVC2*, and the third significant hit was near *DLGAP2*. In FHS, a nearby SNP in *EVC2* showed p ∼ 10^−3^ (Figure S36), and a nearby SNP in *DLGAP2* showed p ∼ 10^−5^.

**Figure 8:**
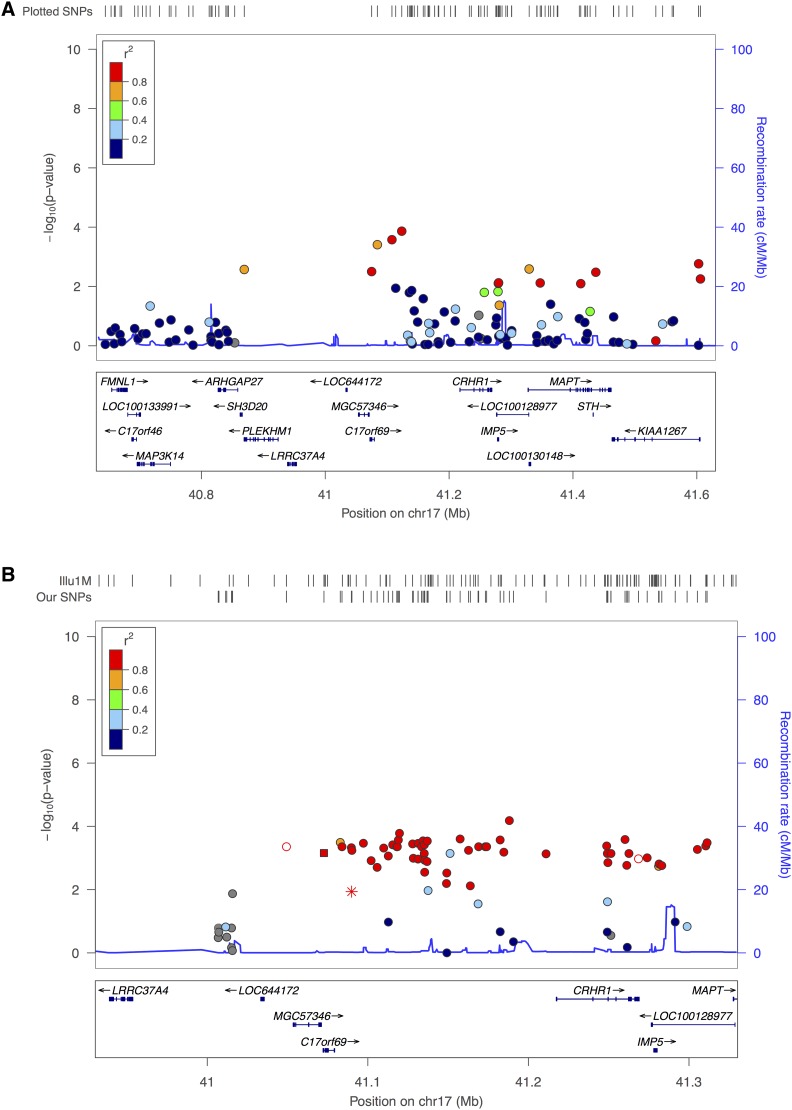
Chr17 inversion region in AGRE/GDCS and FHS female datasets: (A) HS_CNT female in AGRE/GDCS dataset. (B) ARC female in FHS dataset. This figure displays around 1000 and 5000 kb regions around *chr 17* inversion region in three datasets. SNPs in FHS dataset is in high LD compared to two other datasets. The SNPs are color-coded according to HapMap Phase II CEU LD pattern between SNPs (presented in inset in upper right corner). Known genes, and orientation notes are plotted below the SNPs. HapMap recombination rates has been shown with a blue line behind the SNPs. SNP coverage in FHS datasets, and Illumina one million chip is noted by tick marks above the plot.

We also looked at the previously reported genes from Kong *et al.* (2010, 2014), and others in the FHS dataset. For the ARC phenotype, in males *NUB1* (p ∼ 10^−3^), *UGCG* (p ∼ 1.34e^−4^), chr5: rs17542943 (p ∼ 10^−4^), and in females *CRHR1* (p ∼ 10^−4^), *KIAA1462* (p ∼ 10^−3^), *LRRC37A* (p ∼ 10^−3^), *PDZK1* (p ∼ 10^−3^) were well replicated in the FHS dataset. Among the previously reported genes/SNPs for the HS_PCT phenotype, only one SNP (chr18: rs1864309) was replicated with p ∼ 10^−3^. The FHS dataset also showed replication (p ∼ 10^−5^) of association between the chromosome 17 inversion and the ARC phenotype in females, as presented in Figure 8B. A group of SNPs in strong LD across that 900 kb region showed association with the ARC phenotype in females.

### Further dissection of *PRDM9* and *RNF212*

To gain insight into the roles of the previously reported genes influencing recombination rates, we looked at our association results across all five phenotypes.

#### PRDM9:

The *PRDM9* gene association results for different phenotypes are presented in Table 6. We selected the three SNPs with the lowest p values in our study, and examined their p values across all other phenotypes. *PRDM9* showed no evidence of association with the average recombination count and MOTIF phenotypes. In combined analysis, PRDM9 SNPs are significantly associated with HS_PCT (p < 10^−13^), and also with HS_CNT. *PRDM9* SNPs are associated with HS_PCT in both males and females, with similar effect sizes. The male and female effect sizes are also similar for HS_CNT, although the p values were smaller in males. NHS_CNT showed much stronger association (both p value and effect size) in females than in males. Notably, the effect sizes for HS_CNT were in the opposite direction of those for NHS_CNT, suggesting that these *PRDM9* variants are in some sense shifting recombination out of nonhotspot areas, and into hotspot areas, particularly in females. Equivalently, this can be seen as evidence of the existence of a compensatory mechanism that keeps total recombination relatively constant as *PRDM9* increases or decreases hotspot recombination (again primarily in females).

**Table 6:** 3 SNPs (rs2914263 (23488680 bp), rs1874165 (23559104 bp), and rs1603084 (23567950 bp) of PRDM9 gene on Chr 5 association across phenotypes

Phenotype	Analysis Type	SNP	Effect	SE	p Value	Direction
HS_PCT	Combined	*rs2914263*	−0.0268	0.0052	2.174e−07	− − − −
		*rs1874165*	−0.0436	0.0059	2.115e−13	− − − −
		*rs1603084*	−0.0443	0.006	1.20E−13	− − − −
	Female	*rs2914263*	−0.0266	0.0065	4.594e−05	− −
		*rs1874165*	−0.044	0.0076	5.915e−09	− −
		*rs1603084*	−0.0454	0.0076	2.54E−09	− −
	Male	*rs2914263*	−0.0271	0.0084	0.001355	− −
		*rs1874165*	−0.0431	0.0096	7.59E−06	− −
		*rs1603084*	−0.0427	0.0097	9.794e−06	− −
HS_CNT	Combined	*rs2914263*	−0.1301	0.0834	0.1187	− − − −
		*rs1874165*	−0.5237	0.0952	3.80E−08	− − − −
		*rs1603084*	−0.5211	0.0959	5.48e−08	− − − −
	Female	*rs2914263*	−0.1197	0.1357	0.3775	− −
		*rs1874165*	−0.4826	0.1577	0.00221	− −
		*rs1603084*	−0.4849	0.1590	0.002288	− −
	Male	*rs2914263*	−0.1363	0.1057	0.1969	− −
		*rs1874165*	−0.5473	0.1195	4.62E−06	− −
		*rs1603084*	−0.5419	0.1202	6.559e−06	− −
NHS_CNT	Combined	*rs2914263*	0.4931	0.093	1.16E−07	++++
		*rs1874165*	0.4265	0.1075	7.247e−05	++++
		*rs1603084*	0.4381	0.1078	4.792e−05	++++
	Female	*rs2914263*	0.6277	0.1511	3.284e−05	++
		*rs1874165*	0.7679	0.1768	1.406e−05	++
		*rs1603084*	0.8056	0.1784	6.29E−06	++
	Male	*rs2914263*	0.4110	0.1180	0.000498	++
		*rs1874165*	0.2264	0.1354	0.09446	++
		*rs1603084*	0.2269	0.1352	0.09336	++
ARC	Combined	*rs2914263*	0.4920	0.2191	0.02472	++++
		*rs1874165*	−0.0545	0.2531	0.8294	−+−
		*rs1603084*	−0.0332	0.2507	0.8947	−+−
	Female	*rs2914263*	0.5318	0.4582	0.2458	++
		*rs1874165*	0.2609	0.5365	0.6268	−+
		*rs1603084*	0.3037	0.5412	0.5746	−+
	Male	*rs2914263*	0.4802	0.2495	0.05422	++
		*rs1874165*	−0.1448	0.2870	0.6139	–
		*rs1603084*	−0.1253	0.2829	0.6579	–
MOTIF	Combined	*rs2914263*	−0.0033	0.0049	0.4967	+−+−
		*rs1874165*	−0.0084	0.0057	0.1387	−+−
		*rs1603084*	−0.0091	0.0057	0.1141	−+−
	Female	*rs2914263*	0.0018	0.0064	0.7736	++
		*rs1874165*	−0.0126	0.0075	0.09211	–
		*rs1603084*	−0.0138	0.0076	0.06883	–
	Male	*rs2914263*	−0.0108	0.0077	0.16	–
		*rs1874165*	−0.0027	0.0087	0.7561	+−
		*rs1603084*	−0.0027	0.0088	0.7596	+−

#### RNF212:

Table 7 presents the *RNF212* association p values across all phenotypes, though it is primarily associated with ARC phenotype. Females show no association with *RNF212* for any phenotype. In males, *RNF212* SNPs show association with HS_CNT (with p ∼ 10^−5^) but not with NHS_CNT, and only slight association with HS_PCT.

**Table 7:** 2 SNPs (*rs4974601* (1085409 bp) and *rs12645644* (1044158 bp) of *RNF212* gene on Chr 4 association across phenotypes

Analysis type	SNP	ARC		HS_PCT		HS_CNT		NHS_CNT		MOTIF	
		Effect (SE)	p Value	Effect (SE)	p Value	Effect (SE)	p Value	Effect (SE)	p Value	Effect (SE)	p Value
Combined	rs4974601	−0.776(0.15)	2.76E−07	−0.01(0.004)	0.20	−0.21(0.07)	0.001	−0.02(0.07)	0.77	0.01(0.003)	0.06
	rs12645644	−0.64(0.17)	0.0002	−0.002(0.004)	0.59	−0.10(0.06)	0.06	0.01(0.07)	0.88	0.01(0.004)	0.06
Female	rs4974601	0.51(0.37)	0.16	0.002(0.005)	0.64	0.03(0.11)	0.82	−0.02(0.12)	0.84	0.01(0.01)	0.167
	rs12645644	−0.10(0.32)	0.75	0.005(0.005)	0.31	0.09(0.10)	0.35	0.03(0.11)	0.75	0.01(0.005)	0.09
Male	rs4974601	−0.97(0.20)	7.56E−07	−0.02(0.007)	0.007	−0.36(0.08)	2.04e−05	−0.02(0.10)	0.83	0.01(0.01)	0.21
	rs12645644	−0.96(0.17)	1.695e−08	−0.01(0.006)	0.03	−0.22(0.07)	0.002	−0.005(0.08)	0.96	0.01(0.01)	0.34

## Discussion

The goal of this work was to expand our understanding of genetic control of meiotic recombination, finding new recombination genes and more information about already known genes by analyzing new datasets and new phenotypes, particularly phenotypes involving recombination in and out of recognized hotspot regions, and to ask whether the recently discovered recombination genes in the Icelandic population also show association in a United States population.

With regard to the most well-established recombination genes, *RNF212* and *PRDM9*, our results provide new insight into recombination differences between males and females. *RNF212* is well known to affect total recombination, particularly in males, and *PRDM9* is similarly conclusively associated with recombination in hotspots in both males and females, but recombination outside of hotspots has not previously been studied specifically. Kong *et al.* (2014) showed that markers in *PRDM9* are associated with total recombination in males but not females. This suggests that females might have a compensatory mechanism, such that increased recombination in hotspots is balanced by decreased recombination elsewhere. Our results provide further evidence for this hypothesis. In females, we observed that *PRDM9* was associated with both HS_CNT and NHS_CNT, but with effects in opposite directions, which is exactly what would be expected if the hypothesized compensatory mechanism existed. In males, we observed an effect of PRDM9 only on HS_CNT, not NHS_CNT, consistent with the lack of the compensatory mechanism in males. We also observed that markers in *RNF212* are associated with HS_CNT but not NHS_CNT in males, which is again consistent with the idea that males lack such a regulatory mechanism. While far from proof of any hypothesis, these results raise important questions that could be explored further in larger datasets.

We nominated several potential new recombination genes, including a SNP on chromosome 5 (rs12186491) in the protein coding gene *SPINK6*, a serine protease inhibitor, in combined-sex analysis with p = 6.36E−08. Another SNP of interest is chr4: rs10937651, with p = 5.16E−08 in the protein-coding gene *EVC2*, which showed genome-wide significant association with recombination outside of hotspots in males. Two other genes showed lesser statistical significance in our GWAS but replicated in the FHS dataset; *ARHGAP25* (associated with female HS_CNT), and *DLGAP2* (associated with male NHS_CNT). *ARHGAP25* plays role in actin remodeling, cell polarity, and cell migration (Katoh and Katoh 2004). *DLGAP2*, which was associated with recombination in males in our study, is an imprinted gene that is highly expressed in the testes (Luedi *et al.* 2007).

This was also the first study to attempt to replicate the genes found by Kong *et al.* (2014) in the Icelandic population. We conducted our replication at the gene level, in consideration of the significant population and chip differences. We clearly replicated the association near *CPLX1* and *GAK* on chromosome 4 in females. We also replicated their findings on chromosome 14 near *SMEK1* for female recombination. Another association on chromosome 14 from Kong *et al.* (2014) was near C14orf39 in females; we detected only a small signal in females, but a strong association (p < 10−6) in males, a new result that may reflect differences between the Icelandic and United States populations. Other associations from Kong *et al.* (2014) were not replicated in our study, primarily in regions in which our study had poor coverage, or in which the associated variant in Kong *et al.* (2014) was rare. In that sense, we replicated all of the Kong *et al.* (2014) results that we could have expected to, which supports the conclusions of most literature to date that recombination genes tend to have consistent effects across populations.

## Supplementary Material

Supplemental Material

## References

[bib1] Baudat F. Buard J. Grey C. Fledel-Alon A. Ober C., 2010 PRDM9 is a major determinant of meiotic recombination hotspots in humans and mice. Science 327(5967): 836–840. 20044539 10.1126/science.1183439 PMC4295902

[bib2] Begum F. Ghosh D. Tseng G. C. Feingold E., 2012 Comprehensive literature review and statistical considerations for GWAS meta-analysis. Nucleic Acids Res. 40(9): 3777–3784. 22241776 10.1093/nar/gkr1255 PMC3351172

[bib3] Berg I. L. Neumann R. Lam K. W. Sarbajna S. Odenthal-Hesse L., 2010 PRDM9 variation strongly influences recombination hot-spot activity and meiotic instability in humans. Nat. Genet. 42(10): 859–863. 20818382 10.1038/ng.658 PMC3092422

[bib4] Berg I. L. Neumann R. Sarbajna S. Odenthal-Hesse L. Butler N. J., 2011 Variants of the protein PRDM9 differentially regulate a set of human meiotic recombination hotspots highly active in African populations. Proc. Natl. Acad. Sci. USA 108(30): 12378–12383. 21750151 10.1073/pnas.1109531108 PMC3145720

[bib5] Brieno-Enriquez M. A. Cohen P. E., 2015 Double trouble in human aneuploidy. Nat. Genet. 47(7): 696–698. 26111508 10.1038/ng.3344

[bib6] Broman K. W. Murray J. C. Sheffield V. C. White R. L. Weber J. L., 1998 Comprehensive human genetic maps: individual and sex-specific variation in recombination. Am. J. Hum. Genet. 63(3): 861–869. 9718341 10.1086/302011 PMC1377399

[bib7] Cheung V. G. Burdick J. T. Hirschmann D. Morley M., 2007 Polymorphic variation in human meiotic recombination. Am. J. Hum. Genet. 80(3): 526–530. 17273974 10.1086/512131 PMC1821106

[bib8] Cheung V. G. Sherman S. L. Feingold E., 2010 Genetic control of hotspots. Science 327(5967): 791–792. 20150474 10.1126/science.1187155

[bib9] Chowdhury R. Bois P. R. Feingold E. Sherman S. L. Cheung V. G., 2009 Genetic analysis of variation in human meiotic recombination. PLoS Genet. 5(9): e1000648. 19763160 10.1371/journal.pgen.1000648 PMC2730532

[bib10] Coop G. Wen X. Ober C. Pritchard J. K. Przeworski M., 2008 High-resolution mapping of crossovers reveals extensive variation in fine-scale recombination patterns among humans. Science 319(5868): 1395–1398. 18239090 10.1126/science.1151851

[bib11] D’Asdia M. C. Torrente I. Consoli F. Ferese R. Magliozzi M., 2013 Novel and recurrent EVC and EVC2 mutations in Ellis-van Creveld syndrome and Weyers acrofacial dyostosis. Eur. J. Med. Genet. 56(2): 80–87. 23220543 10.1016/j.ejmg.2012.11.005

[bib12] Dawber T. R. Meadors G. F. Moore F. E. Jr, 1951 Epidemiological approaches to heart disease: the Framingham study. Am. J. Public Health Nations Health 41(3): 279–281. 14819398 10.2105/ajph.41.3.279 PMC1525365

[bib13] Fledel-Alon A. Leffler E. M. Guan Y. Stephens M. Coop G., 2011 Variation in human recombination rates and its genetic determinants. PLoS One 6(6): e20321. 21698098 10.1371/journal.pone.0020321 PMC3117798

[bib14] Hinch A. G. Tandon A. Patterson N. Song Y. Rohland N., 2011 The landscape of recombination in African Americans. Nature 476(7359): 170–175. 21775986 10.1038/nature10336 PMC3154982

[bib15] Hou Y. Fan W. Yan L. Li R. Lian Y., 2013 Genome analyses of single human oocytes. Cell 155(7): 1492–1506. 24360273 10.1016/j.cell.2013.11.040

[bib16] International HapMap Consortium Frazer K. A. Ballinger D. G. Cox D. R. Hinds D. A. Stuve L. L., 2007 A second generation human haplotype map of over 3.1 million SNPs. Nature 449(7164): 851–861. 17943122 10.1038/nature06258 PMC2689609

[bib17] Kamal R. Dahiya P. Kaur S. Bhardwaj R. Chaudhary K., 2013 Ellis-van Creveld syndrome: a rare clinical entity. J. Oral Maxillofac. Pathol. 17(1): 132–135. 23798848 10.4103/0973-029X.110716 PMC3687170

[bib18] Katoh M. Katoh M., 2004 Identification and characterization of ARHGAP24 and ARHGAP25 genes in silico. Int. J. Mol. Med. 14(2): 333–338. 15254788

[bib19] Kauppi L. Jeffreys A. J. Keeney S., 2004 Where the crossovers are: recombination distributions in mammals. Nat. Rev. Genet. 5(6): 413–424. 15153994 10.1038/nrg1346

[bib20] Kimura K. Wakamatsu A. Suzuki Y. Ota T. Nishikawa T., 2006 Diversification of transcriptional modulation: large-scale identification and characterization of putative alternative promoters of human genes. Genome Res. 16(1): 55–65. 16344560 10.1101/gr.4039406 PMC1356129

[bib21] Kong A. Thorleifsson G. Stefansson H. Masson G. Helgason A., 2008 Sequence variants in the RNF212 gene associate with genome-wide recombination rate. Science 319(5868): 1398–1401. 18239089 10.1126/science.1152422

[bib22] Kong A. Thorleifsson G. Gudbjartsson D. F. Masson G. Sigurdsson A., 2010 Fine-scale recombination rate differences between sexes, populations and individuals. Nature 467(7319): 1099–1103. 20981099 10.1038/nature09525

[bib23] Kong A. Thorleifsson G. Frigge M. L. Masson G. Gudbjartsson D. F., 2014 Common and low-frequency variants associated with genome-wide recombination rate. Nat. Genet. 46(1): 11–16. 24270358 10.1038/ng.2833

[bib24] Lin D. Y. Zeng D., 2010 Meta-analysis of genome-wide association studies: no efficiency gain in using individual participant data. Genet. Epidemiol. 34(1): 60–66. 19847795 10.1002/gepi.20435 PMC3878085

[bib25] Luedi P. P. Dietrich F. S. Weidman J. R. Bosko J. M. Jirtle R. L., 2007 Computational and experimental identification of novel human imprinted genes. Genome Res. 17(12): 1723–1730. 18055845 10.1101/gr.6584707 PMC2099581

[bib26] MacLennan M. Crichton J. H. Playfoot C. J. Adams I. R., 2015 Oocyte development, meiosis and aneuploidy. Semin. Cell Dev. Biol. 5:68–76. 10.1016/j.semcdb.2015.10.005 PMC4828587 26454098

[bib27] Myers S. Bottolo L. Freeman C. McVean G. Donnelly P., 2005 A fine-scale map of recombination rates and hotspots across the human genome. Science 310(5746): 321–324. 16224025 10.1126/science.1117196

[bib28] Myers S. Freeman C. Auton A. Donnelly P. McVean G., 2008 A common sequence motif associated with recombination hot spots and genome instability in humans. Nat. Genet. 40(9): 1124–1129. 19165926 10.1038/ng.213

[bib29] Neale M. J., 2010 PRDM9 points the zinc finger at meiotic recombination hotspots. Genome Biol. 11(2): 104. 20210982 10.1186/gb-2010-11-2-104 PMC2872867

[bib30] Pruim R. J. Welch R. P. Sanna S. Teslovich T. M. Chines P. S., 2010 LocusZoom: regional visualization of genome-wide association scan results. Bioinformatics 26(18): 2336–2337. 20634204 10.1093/bioinformatics/btq419 PMC2935401

[bib31] Sandovici I. Sapienza C., 2010 PRDM9 sticks its zinc fingers into recombination hotspots and between species. F1000 Biol Rep. 2: 37. 20948797 10.3410/B2-37 PMC2950028

[bib32] Sarbajna S. Denniff M. Jeffreys A. J. Neumann R. Artigas M. S., 2012 A major recombination hotspot in the XqYq pseudoautosomal region gives new insight into processing of human gene conversion events. Hum. Mol. Genet. 21(9):2029–2038. 22291443 10.1093/hmg/dds019

[bib33] Segurel L. Leffler E. M. Przeworski M., 2011 The case of the fickle fingers: how the PRDM9 zinc finger protein specifies meiotic recombination hotspots in humans. PLoS Biol. 9(12): e1001211. 22162947 10.1371/journal.pbio.1001211 PMC3232208

[bib34] Shaffer J. R. Wang X. Feingold E. Lee M. Begum F., 2011 Genome-wide association scan for childhood caries implicates novel genes. J. Dent. Res. 90(12): 1457–1462. 21940522 10.1177/0022034511422910 PMC3215757

[bib35] Sung Y. J. Schwander K. Arnett D. K. Kardia S. L. Rankinen T., 2014 An empirical comparison of meta-analysis and mega-analysis of individual participant data for identifying gene-environment interactions. Genet. Epidemiol. 38(4): 369–378. 24719363 10.1002/gepi.21800 PMC4332385

[bib36] Weiss L. A. Shen Y. Korn J. M. Arking D. E. Miller D. T., 2008 Association between microdeletion and microduplication at 16p11.2 and autism. N. Engl. J. Med. 358(7): 667–675. 18184952 10.1056/NEJMoa075974

[bib37] Willer C. J. Li Y. Abecasis G. R., 2010 METAL: fast and efficient meta-analysis of genomewide association scans. Bioinformatics 26(17): 2190–2191. 20616382 10.1093/bioinformatics/btq340 PMC2922887

[bib38] Yang P. Wu M. Guo J. Kwoh C. K. Przytycka T. M., 2014 LDsplit: screening for cis-regulatory motifs stimulating meiotic recombination hotspots by analysis of DNA sequence polymorphisms. BMC Bioinformatics 15: 48. 24533858 10.1186/1471-2105-15-48 PMC3936957

