# Supplementary material for: Genome-Wide Association Study of Meiotic Recombination Phenotypes
Source: G3 (Bethesda). 2016 Oct 12;6(12):3995–4007. doi: 10.1534/g3.116.035766 (PMC5144969; doi:10.1534/g3.116.035766)
Supplement: Supplemental Material [file supp_6_12_3995__index.html]

Genome-Wide Association Study of Meiotic Recombination Phenotypes — Supplemental Material 

# Genome-Wide Association Study of Meiotic Recombination Phenotypes

## Supplemental Material for Begum *et al.*, 2016

**Files in this Data Supplement:**

- Figure S1 - ARC: Manhattan plot of female meta-analysis (.pdf, 105 KB)
- Figure S2 - ARC: QQ plot of female meta-analysis (.pdf, 28 KB)
- Figure S3 - ARC: Manhattan plot of combined-sex meta-analysis (.pdf, 90 KB)
- Figure S4 - ARC: QQ plot of combined-sex meta-analysis (.pdf, 25 KB)
- Figure S5 - HS\_PCT: Distribution of HS\_PCT (.pdf, 62 KB)
- Figure S6 - HS\_PCT: Manhattan plot of female meta-analysis (.pdf, 92 KB)
- Figure S7 - HS\_PCT: QQ plot of female meta-analysis (.pdf, 26 KB)
- Figure S8 - HS\_PCT: Manhattan plot of male meta-analysis (.pdf, 88 KB)
- Figure S9 - HS\_PCT: QQ plot of male meta-analysis (.pdf, 27 KB)
- Figure S10 - HS\_CNT: Distribution of HS\_CNT phenotype (.pdf, 48 KB)
- Figure S11 - HS\_CNT: Manhattan plot of female meta-analysis (.pdf, 46 KB)
- Figure S32 - Locus zoom plot of previously reported male genes (.pdf, 159 KB)
- Figure S12 - HS\_CNT: QQ plot of female meta-analysis (.pdf, 28 KB)
- Figure S13 - HS\_CNT: Manhattan plot of male meta-analysis (.pdf, 99 KB)
- Figure S14 - HS\_CNT: QQ plot of male meta-analysis (.pdf, 27 KB)
- Figure S15 - HS\_CNT: Manhattan plot of combined-sex meta-analysis (.pdf, 116 KB)
- Figure S16 - HS\_ CNT: QQ plot of combined-sex meta-analysis (.pdf, 31 KB)
- Figure S17 - NHS\_CNT: Distribution of NHS-CNT phenotype in three data sets (.pdf, 46 KB)
- Figure S18 - NHS\_CNT: Manhattan plot of female meta-analysis (.pdf, 104 KB)
- Figure S19 - NHS\_CNT: QQ plot of female meta-analysis (.pdf, 29 KB)
- Figure S20 - NHS\_CNT: Manhattan plot of male meta-analysis (.pdf, 108 KB)
- Figure S21 - NHS\_CNT: QQ plot of male meta-analysis (.pdf, 32 KB)
- Figure S33 - Locus zoom plot of previously reported female genes (.pdf, 157 KB)
- Figure S22 - NHS\_CNT: Manhattan plot of combined-sex meta-analysis (.pdf, 144 KB)
- Figure S23 - NHS\_ CNT: QQ plot of combined-sex meta-analysis (.pdf, 39 KB)
- Figure S24 - MOTIF: Distribution of Motif phenotype in three data sets. (.pdf, 68 KB)
- Figure S25 - MOTIF: Manhattan plot of female meta-analysis (.pdf, 101 KB)
- Figure S26 - MOTIF: QQ plot of female meta-analysis (.pdf, 29 KB)
- Figure S27 - MOTIF: Manhattan plot of male meta-analysis (.pdf, 104 KB)
- Figure S28 - MOTIF: QQ plot of male meta-analysis (.pdf, 31 KB)
- Figure S29 - MOTIF: Manhattan plot of combined-sex meta-analysis (.pdf, 98 KB)
- Figure S30 - MOTIF: QQ plot of combined-sex meta-analysis (.pdf, 45 KB)
- Figure S31 - Locus zoom plot of chr 17 inversion region (.pdf, 98 KB)
- Figure S34 - Locus zoom plot of *Kong et al.* top hits (.pdf, 150 KB)
- Figure S35 - Locus zoom plot of *SPINKS6* in FHS study for NHS\_CNT (.pdf, 68 KB)
- Figure S36 - Locus zoom plot of *EVC2* in FHS study for NHS\_CNT (.pdf, 112 KB)
- Table S1 - SNPs with lowest *p*-values for MOTIF Type (.pdf, 78 KB)
- Table S2 - SNPs with lowest *p*-values for phenotype ARC in FHS (.pdf, 98 KB)
- Table S3 - Replication of Kong *et al.* (2014) findings (.pdf, 95 KB)
