# Supplementary figures and images for: Genome-Wide Association Study of Meiotic Recombination Phenotypes
Source: G3 (Bethesda). 2016 Oct 12;6(12):3995–4007. doi: 10.1534/g3.116.035766 (PMC5144969; doi:10.1534/g3.116.035766)

S1. ARC: Manhattan plot of female meta-analysis

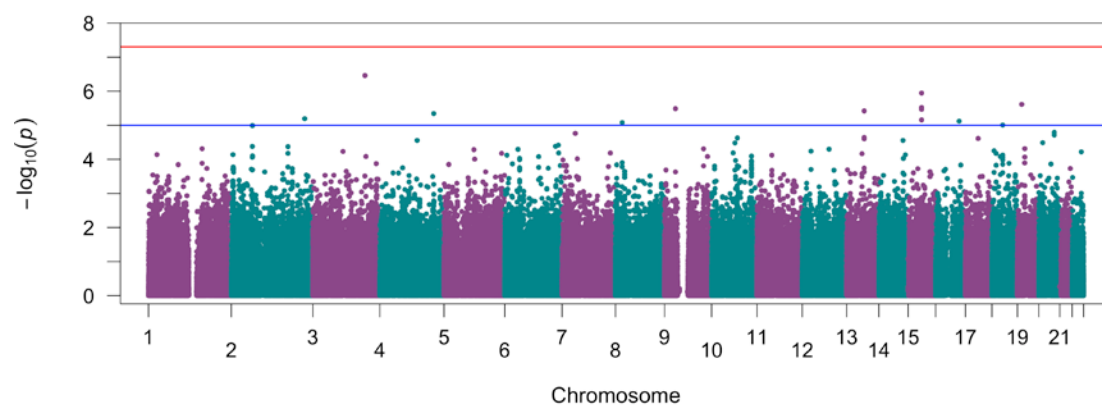

Supplement: Supplemental Material [file supp_g3.116.035766_FigureS1.pdf]

S2. ARC: QQ plot of female meta-analysis

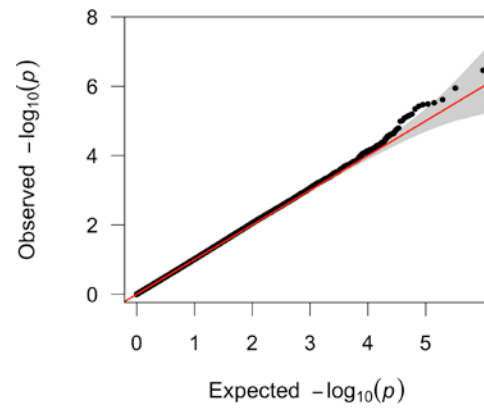

Supplement: Supplemental Material [file supp_g3.116.035766_FigureS2.pdf]

S3.     ARC: Manhattan plot of combined-sex meta-analysis

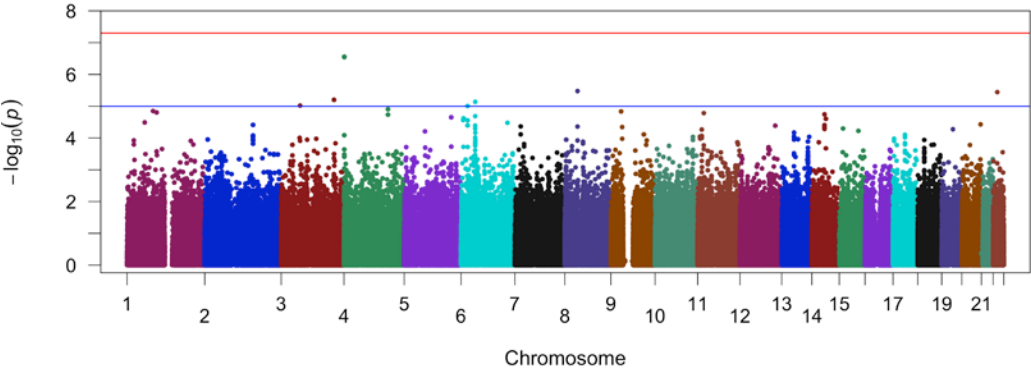

Supplement: Supplemental Material [file supp_g3.116.035766_FigureS3.pdf]

S4. ARC: QQ plot of combined-sex meta-analysis

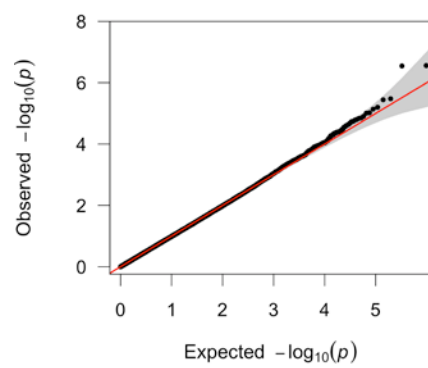

Supplement: Supplemental Material [file supp_g3.116.035766_FigureS4.pdf]

S5. HS\_PCT: Distribution of HS\_PCT

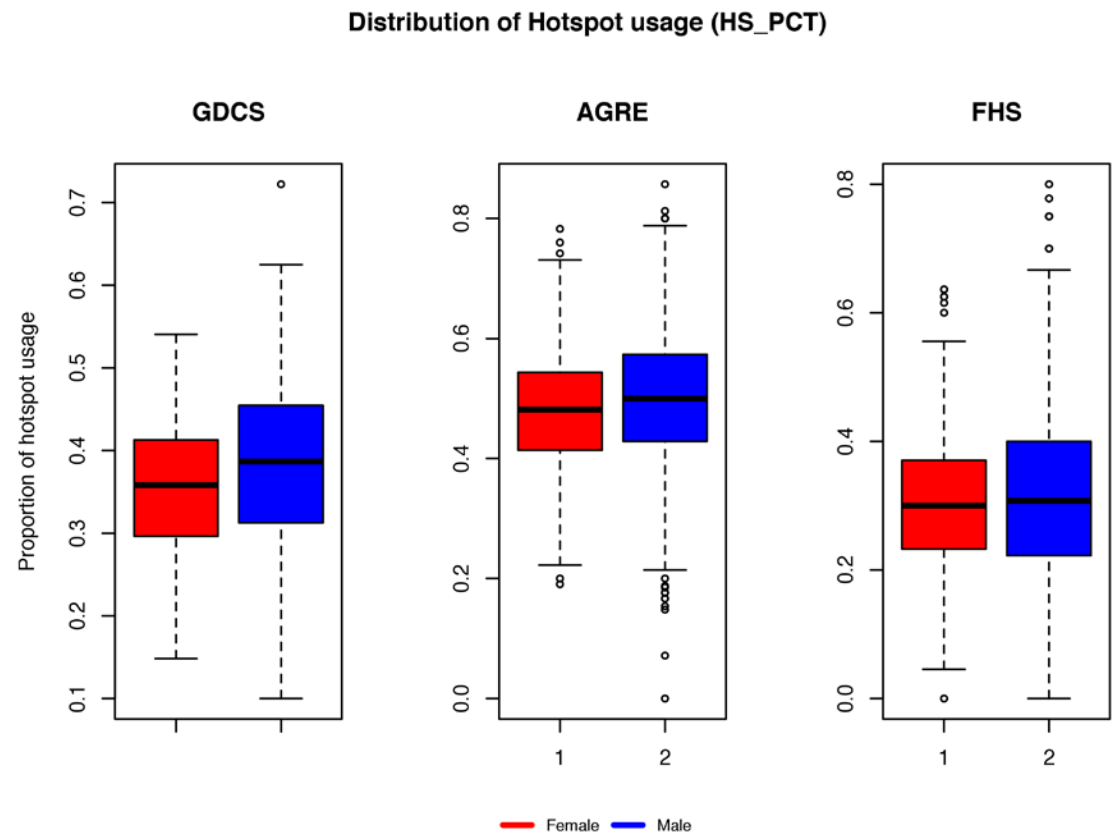

Supplement: Supplemental Material [file supp_g3.116.035766_FigureS5.pdf]

S6. HS\_PCT: Manhattan plot of female meta-analysis

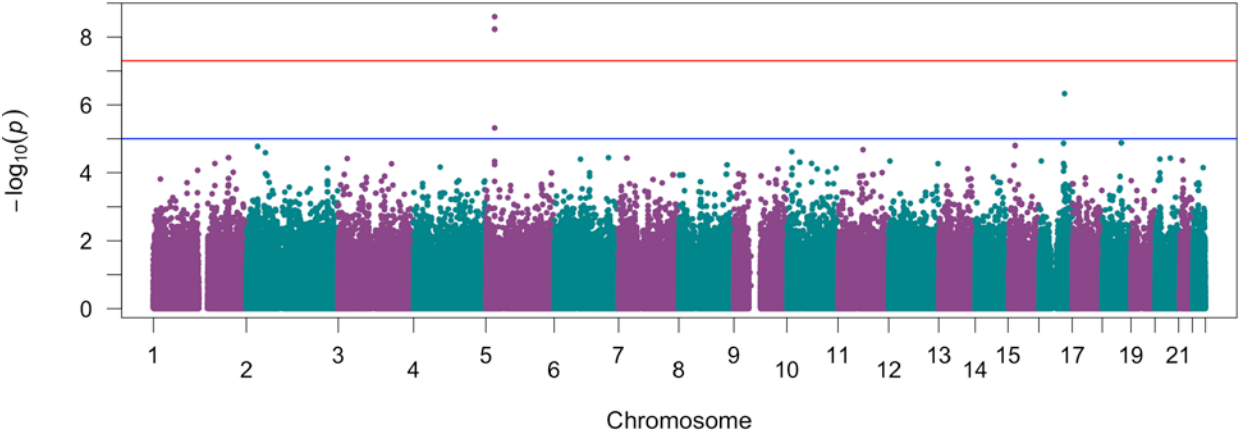

Supplement: Supplemental Material [file supp_g3.116.035766_FigureS6.pdf]

S7. HS\_PCT: QQ plot of female meta-analysis

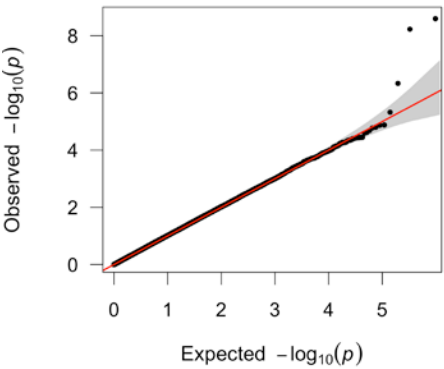

Supplement: Supplemental Material [file supp_g3.116.035766_FigureS7.pdf]

S8. HS\_PCT: Manhattan plot of male meta-analysis

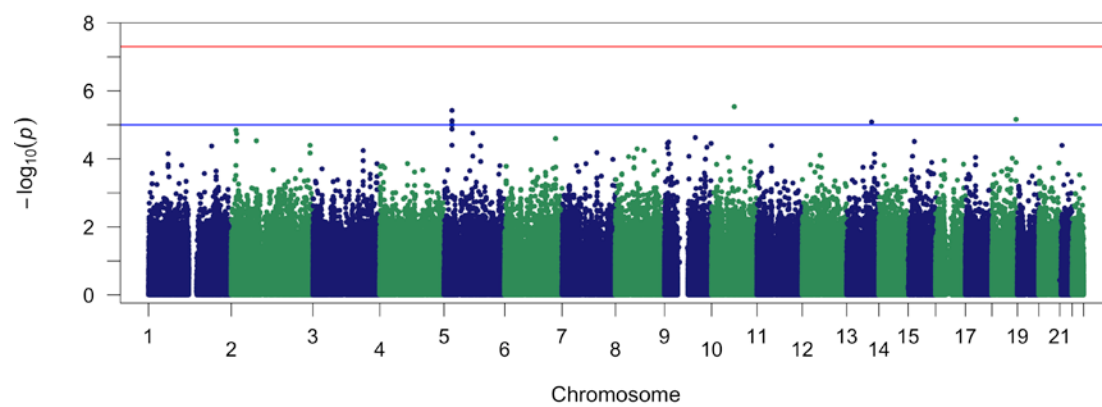

Supplement: Supplemental Material [file supp_g3.116.035766_FigureS8.pdf]

S9. HS\_PCT: QQ plot of male meta-analysis

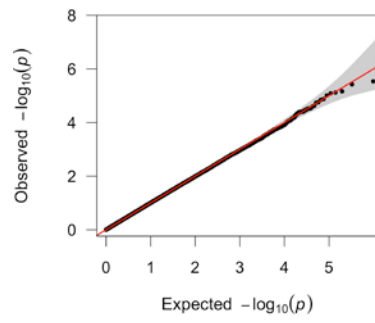

Supplement: Supplemental Material [file supp_g3.116.035766_FigureS9.pdf]

S10. HS\_CNT: Distribution of HS\_CNT phenotype

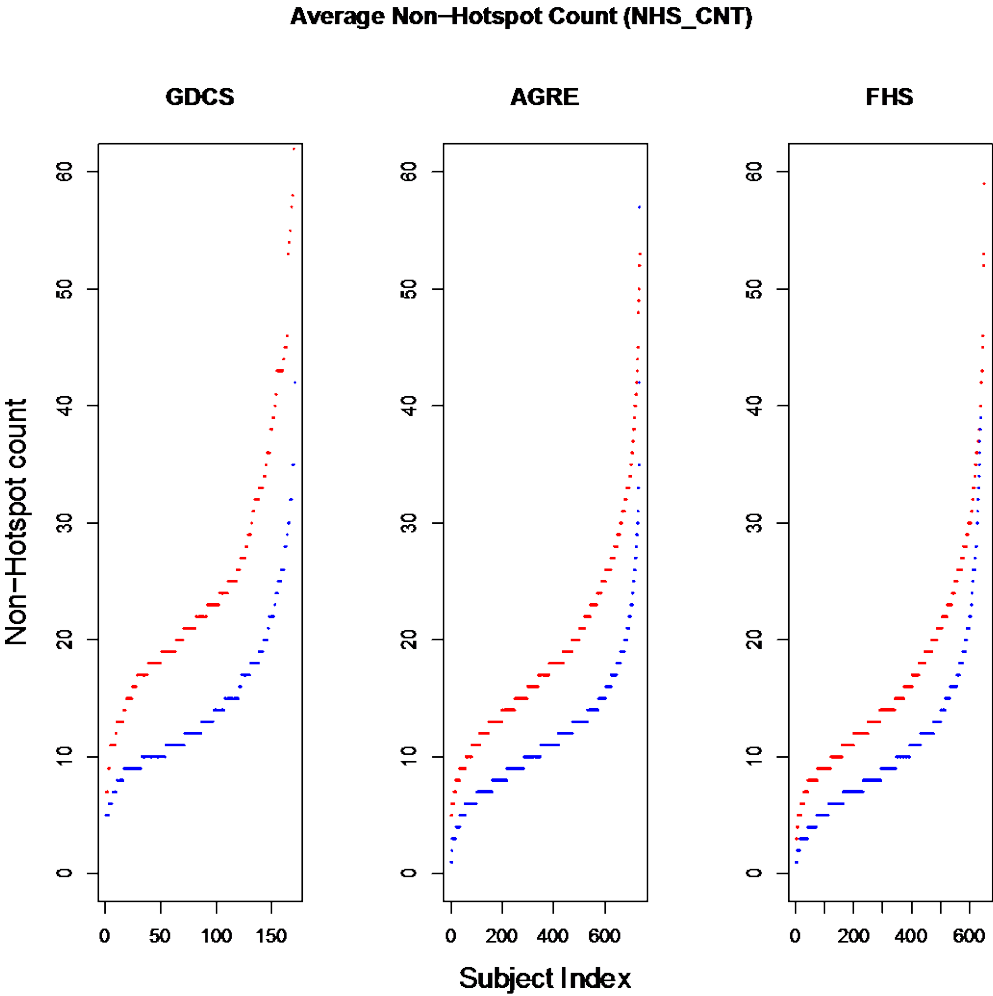

Supplement: Supplemental Material [file supp_g3.116.035766_FigureS10.pdf]

S11. HS\_CNT: Manhattan plot of female meta-analysis

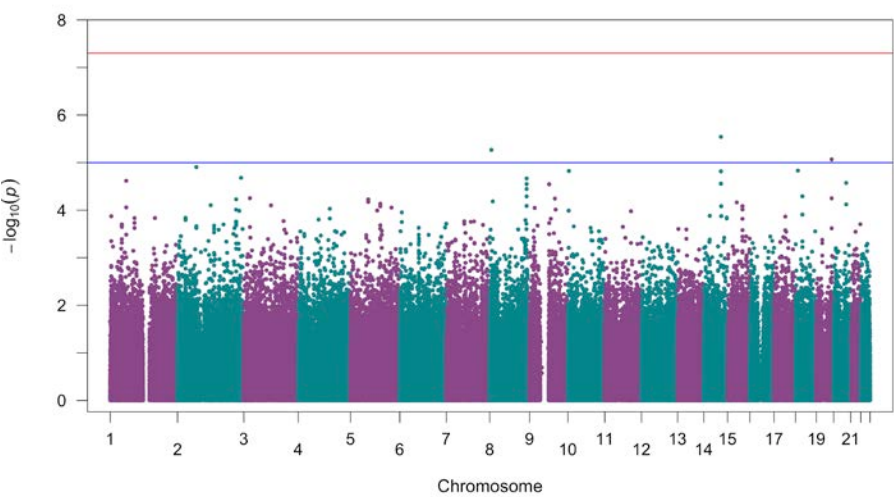

Supplement: Supplemental Material [file supp_g3.116.035766_FigureS11.pdf]

S32. Locus zoom plot of chr 17 inversion region

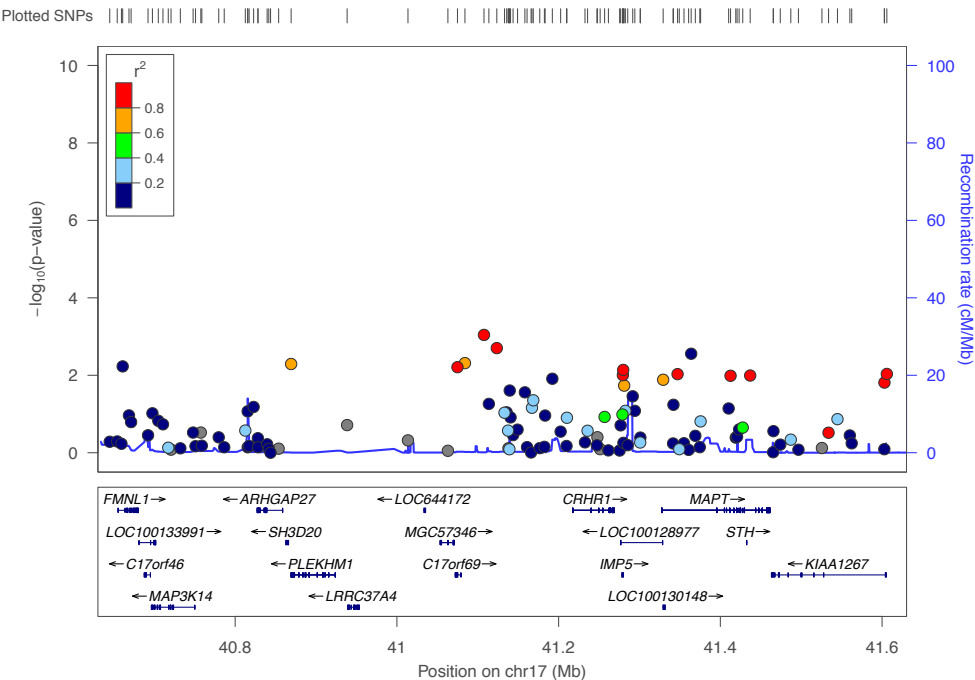

a). ARC (female)

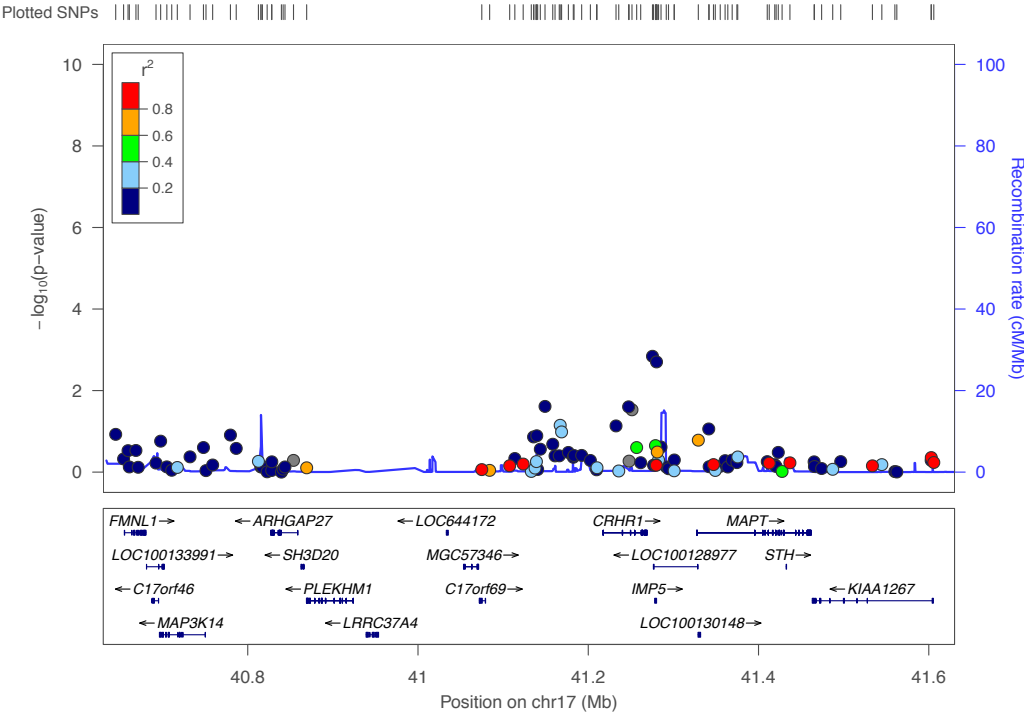

b). MOTIF (Female)

Supplement: Supplemental Material [file supp_g3.116.035766_FigureS32.pdf]

S12. HS\_CNT: QQ plot of female meta-analysis

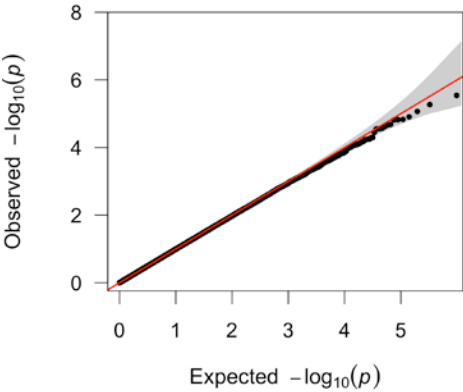

Supplement: Supplemental Material [file supp_g3.116.035766_FigureS12.pdf]

S13. HS\_CNT: Manhattan plot of male meta-analysis

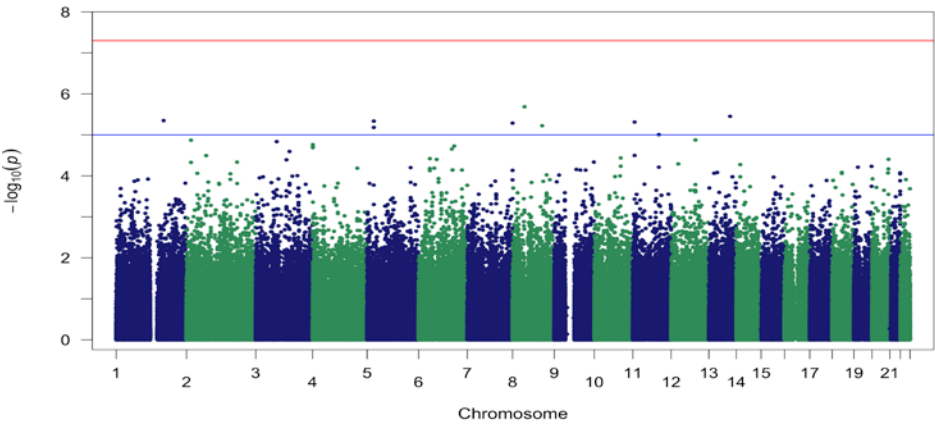

Supplement: Supplemental Material [file supp_g3.116.035766_FigureS13.pdf]

S14. HS\_CNT: QQ plot of male meta-analysis

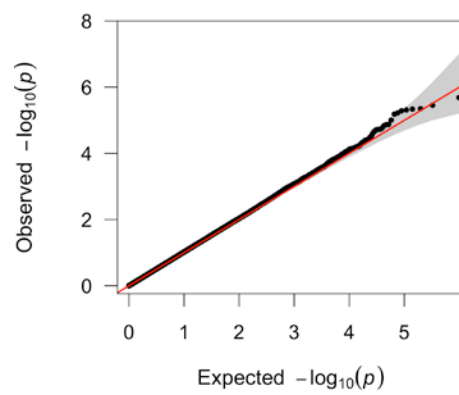

Supplement: Supplemental Material [file supp_g3.116.035766_FigureS14.pdf]

S15. HS\_CNT: Manhattan plot of combined-sex meta-analysis

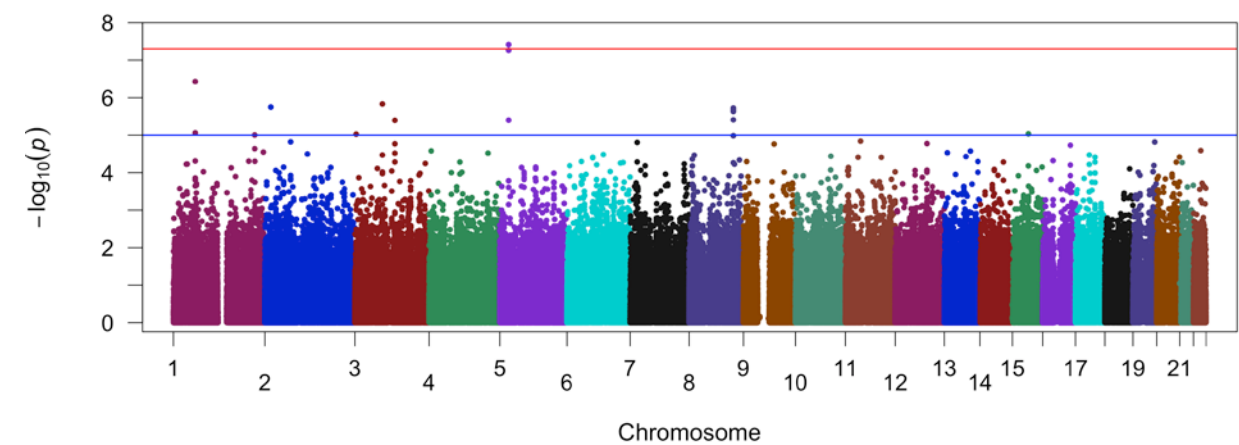

Supplement: Supplemental Material [file supp_g3.116.035766_FigureS15.pdf]

S16. HS\_CNT: QQ plot of combined-sex meta-analysis

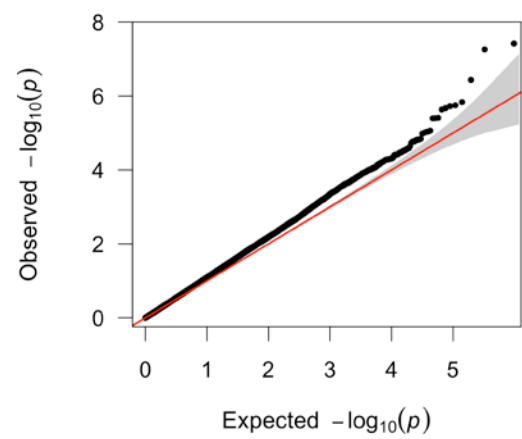

Supplement: Supplemental Material [file supp_g3.116.035766_FigureS16.pdf]

S17. NHS\_CNT: Distribution of NHS-CNT phenotype in three data sets.

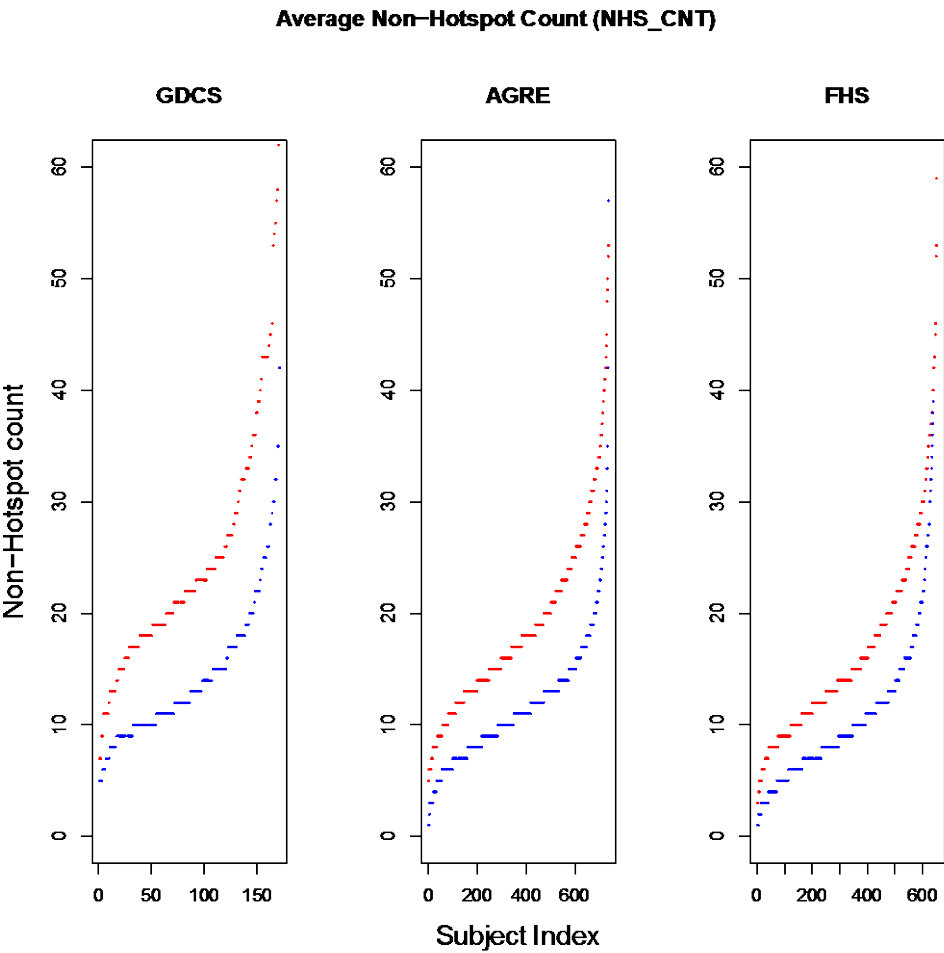

Supplement: Supplemental Material [file supp_g3.116.035766_FigureS17.pdf]

S18. NHS\_CNT: Manhattan plot of female meta-analysis

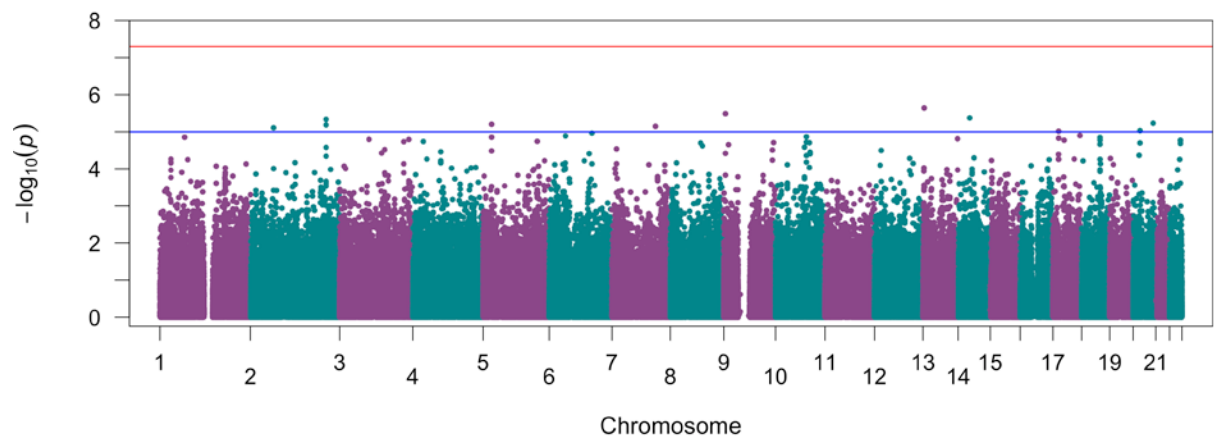

Supplement: Supplemental Material [file supp_g3.116.035766_FigureS18.pdf]

S19. NHS\_CNT: QQ plot of female meta-analysis

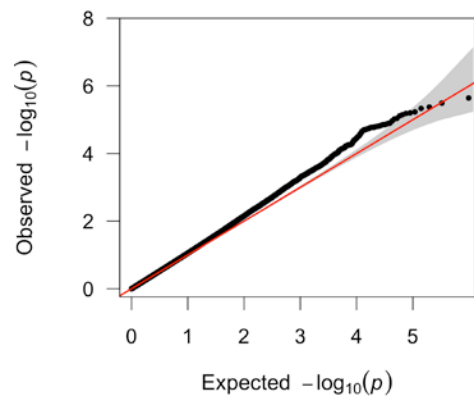

Supplement: Supplemental Material [file supp_g3.116.035766_FigureS19.pdf]

S20. NHS\_CNT: Manhattan plot of male meta-analysis

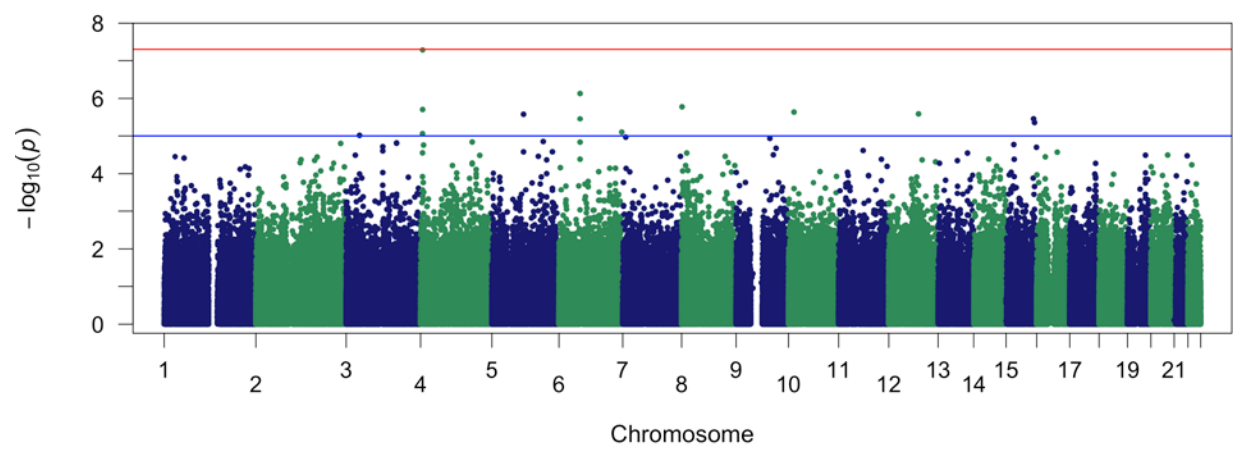

Supplement: Supplemental Material [file supp_g3.116.035766_FigureS20.pdf]

S21. NHS\_CNT: QQ plot of male meta-analysis

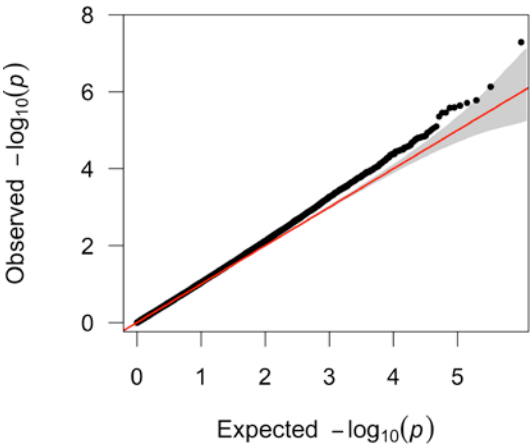

Supplement: Supplemental Material [file supp_g3.116.035766_FigureS21.pdf]

S33. Locus zoom plot of previously reported male genes

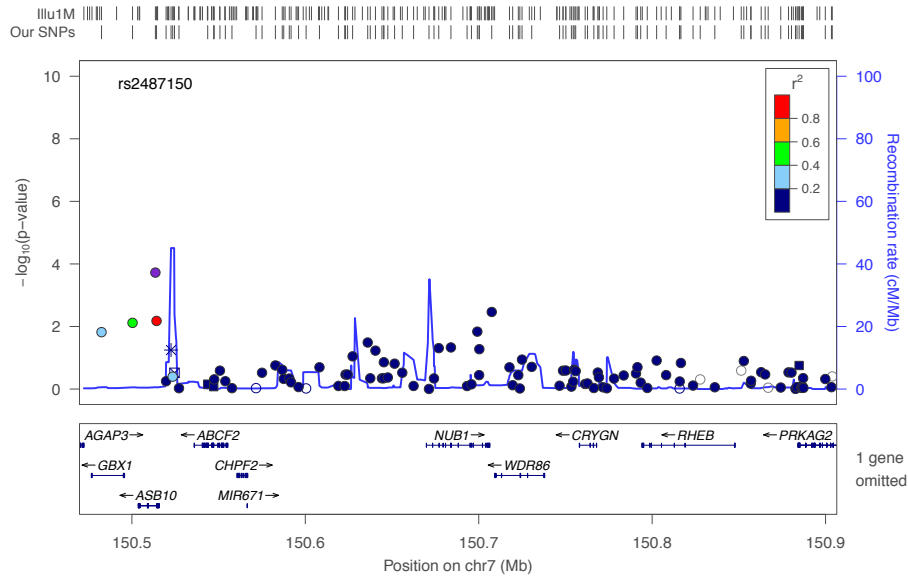

a). *NUB1*

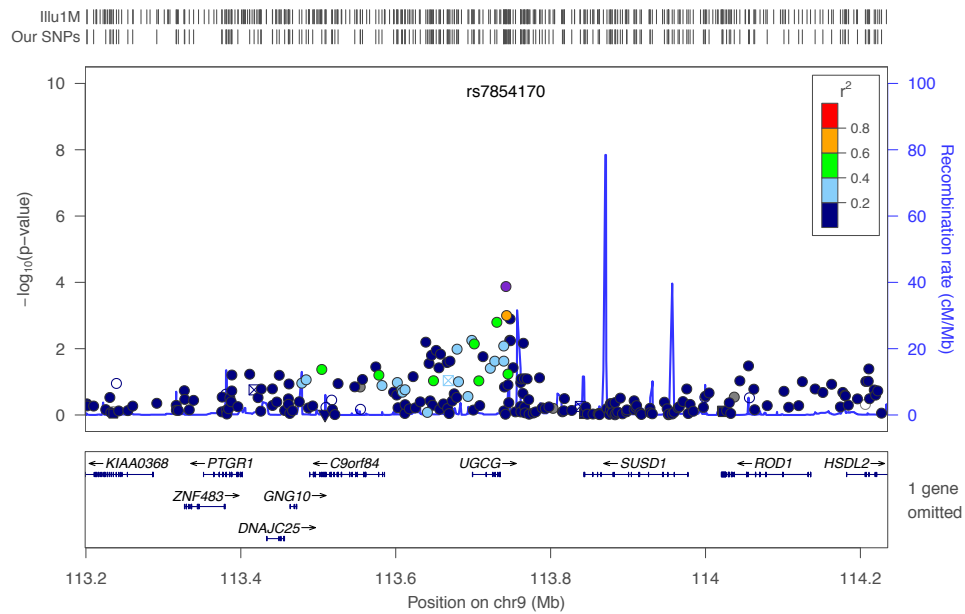

b). *UGCG*

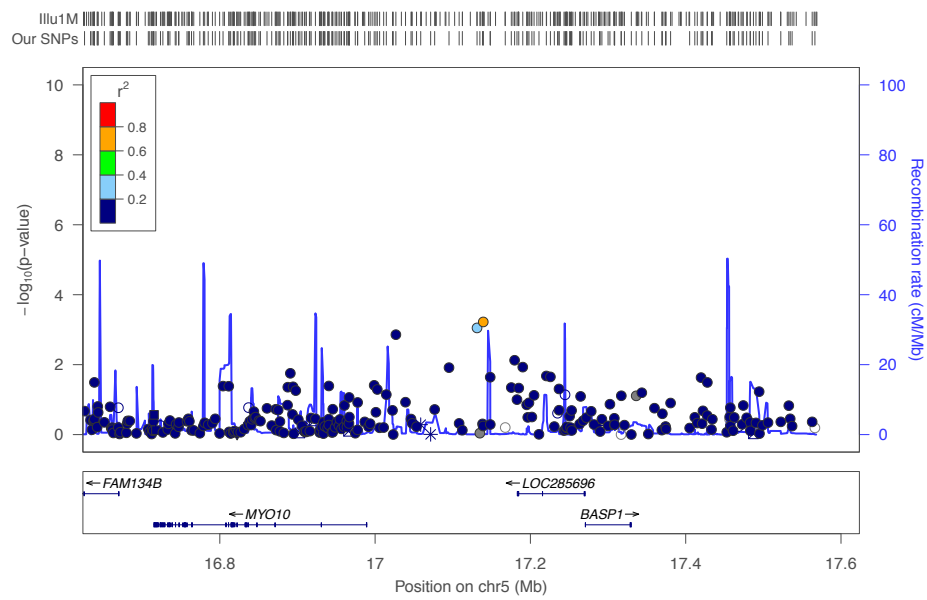

c). SNP (chr5: *rs17542943*).

Supplement: Supplemental Material [file supp_g3.116.035766_FigureS33.pdf]

S22. NHS\_CNT: Manhattan plot of combined-sex meta-analysis

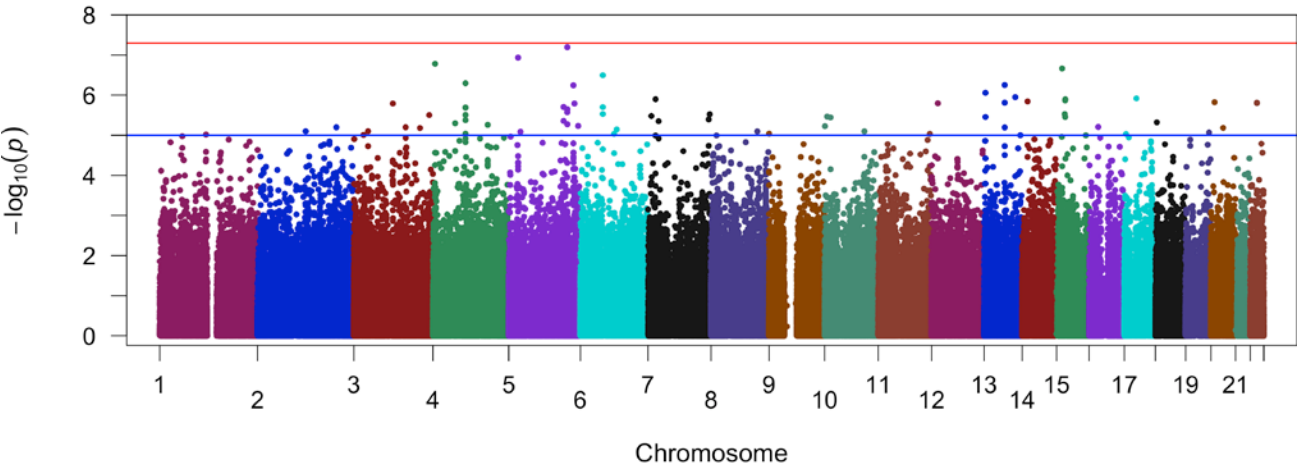

Supplement: Supplemental Material [file supp_g3.116.035766_FigureS22.pdf]

S23. NHS\_ CNT: QQ plot of combined-sex meta-analysis

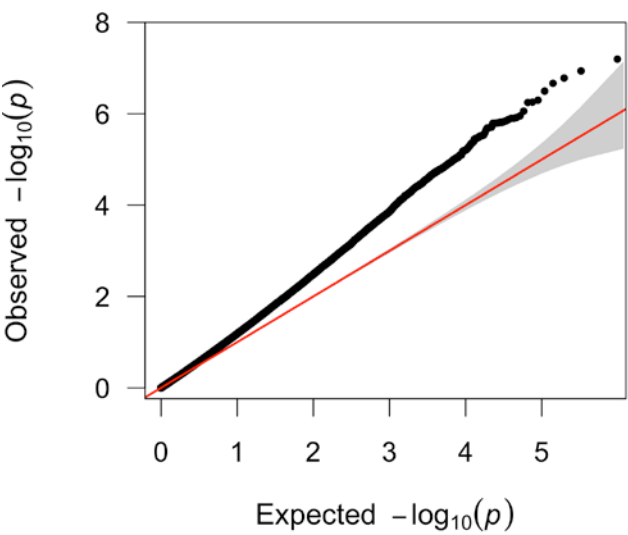

Supplement: Supplemental Material [file supp_g3.116.035766_FigureS23.pdf]

S24. MOTIF: Distribution of Motif phenotype in three data sets.

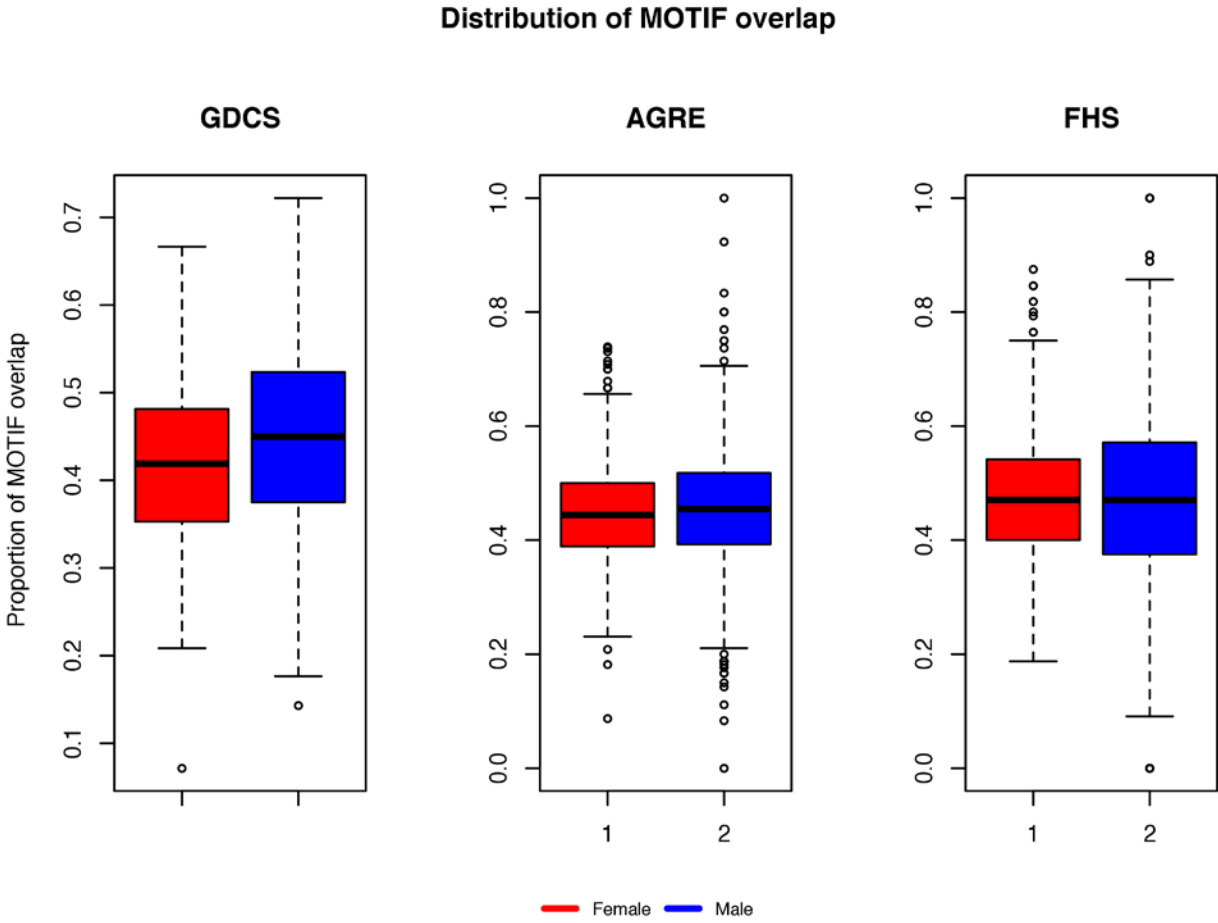

Supplement: Supplemental Material [file supp_g3.116.035766_FigureS24.pdf]

S25. MOTIF: Manhattan plot of female meta-analysis

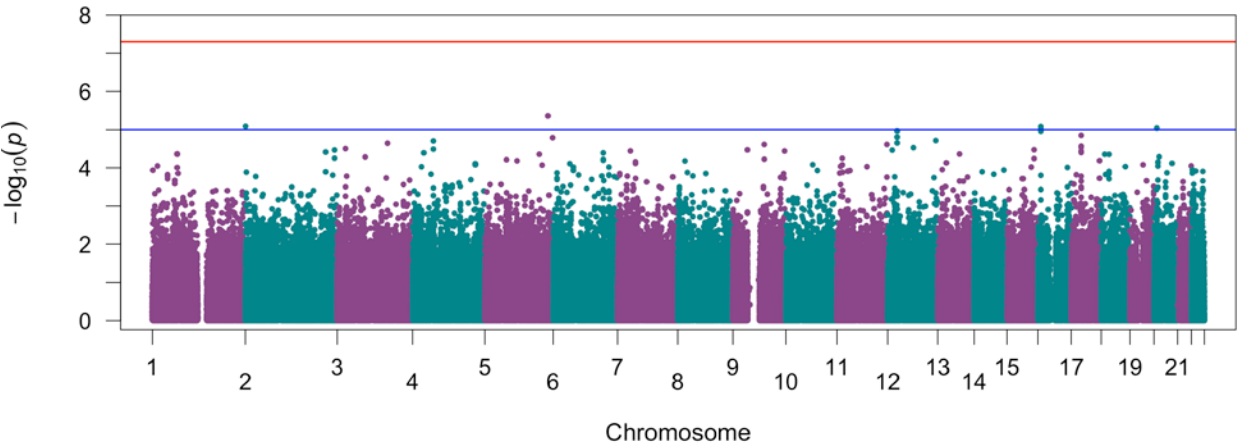

Supplement: Supplemental Material [file supp_g3.116.035766_FigureS25.pdf]

S26. MOTIF: QQ plot of female meta-analysis

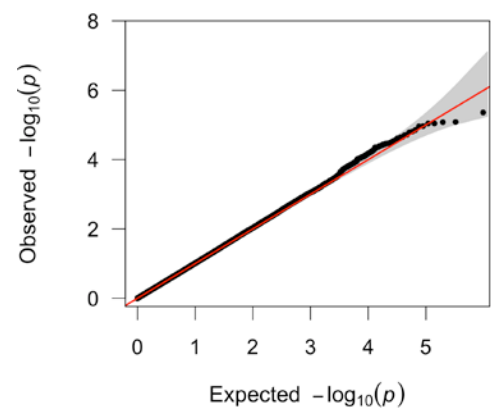

Supplement: Supplemental Material [file supp_g3.116.035766_FigureS26.pdf]

S27. MOTIF: Manhattan plot of male meta-analysis

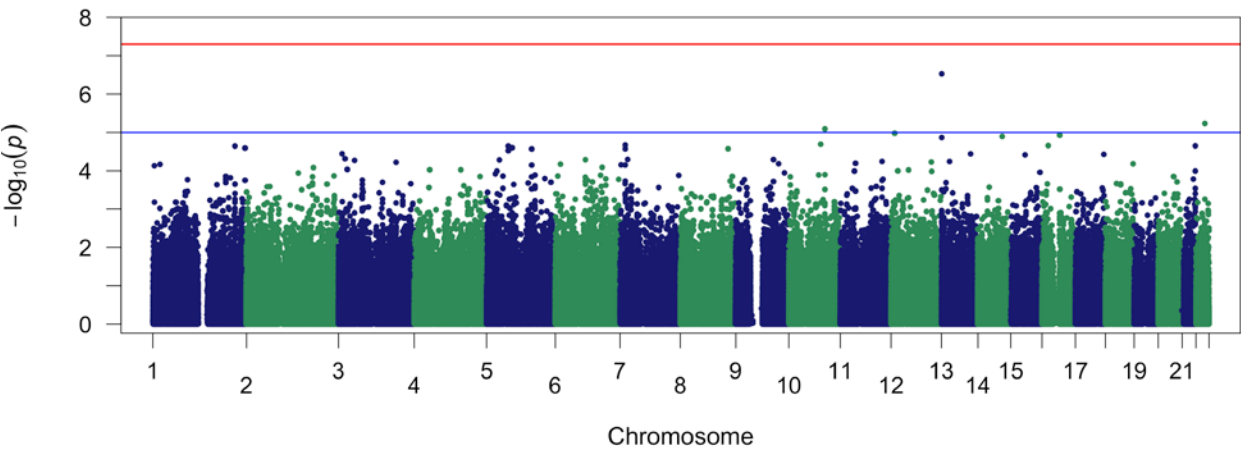

Supplement: Supplemental Material [file supp_g3.116.035766_FigureS27.pdf]

S28. MOTIF: QQ plot of male meta-analysis

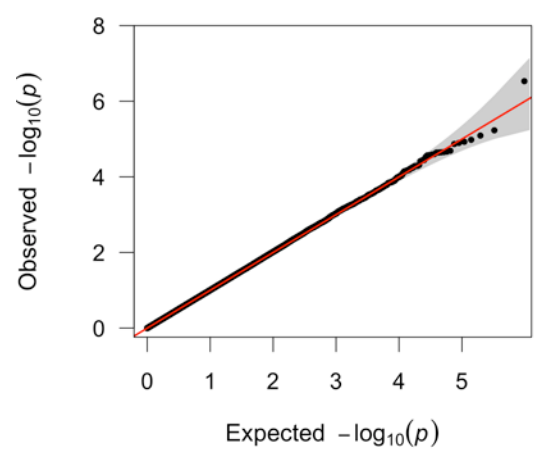

Supplement: Supplemental Material [file supp_g3.116.035766_FigureS28.pdf]

S29. MOTIF: Manhattan plot of combined-sex meta-analysis

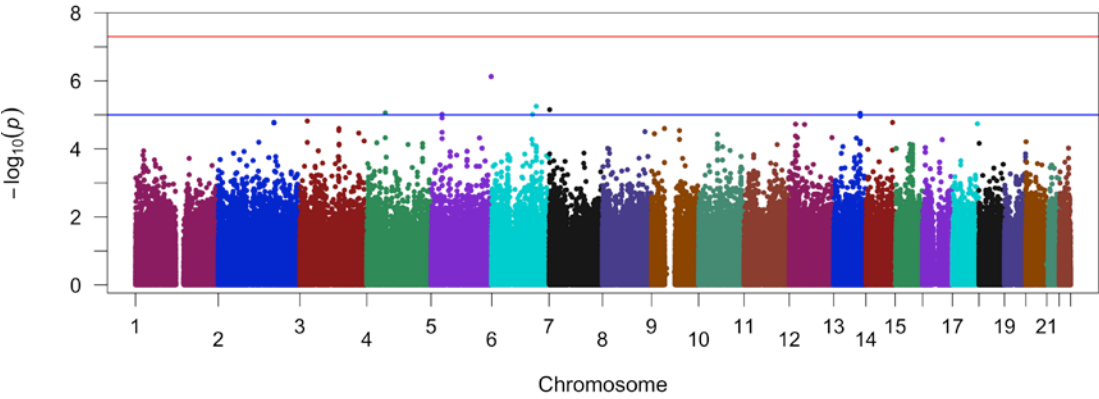

Supplement: Supplemental Material [file supp_g3.116.035766_FigureS29.pdf]

S30. MOTIF: QQ plot of combined-sex meta-analysis

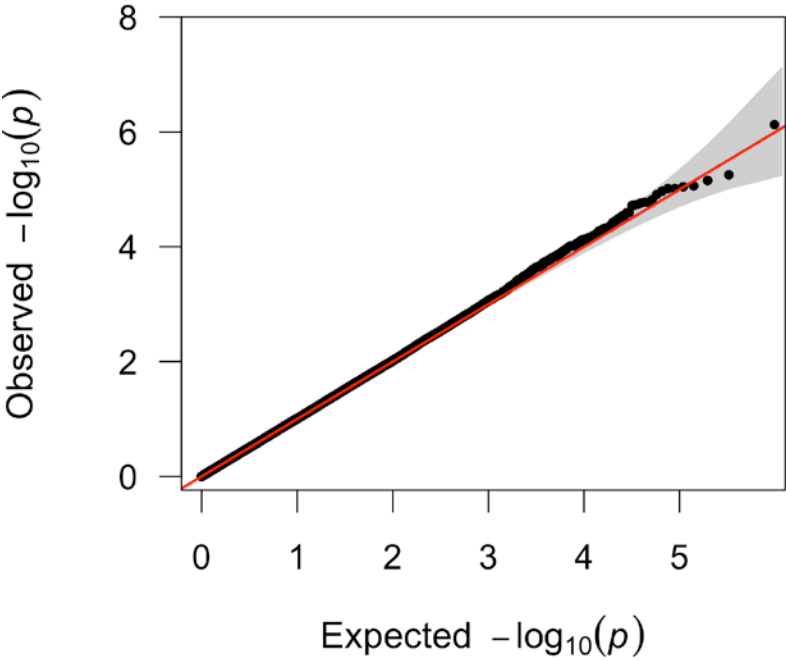

Supplement: Supplemental Material [file supp_g3.116.035766_FigureS30.pdf]

S34. Locus zoom plot of previously reported female genes

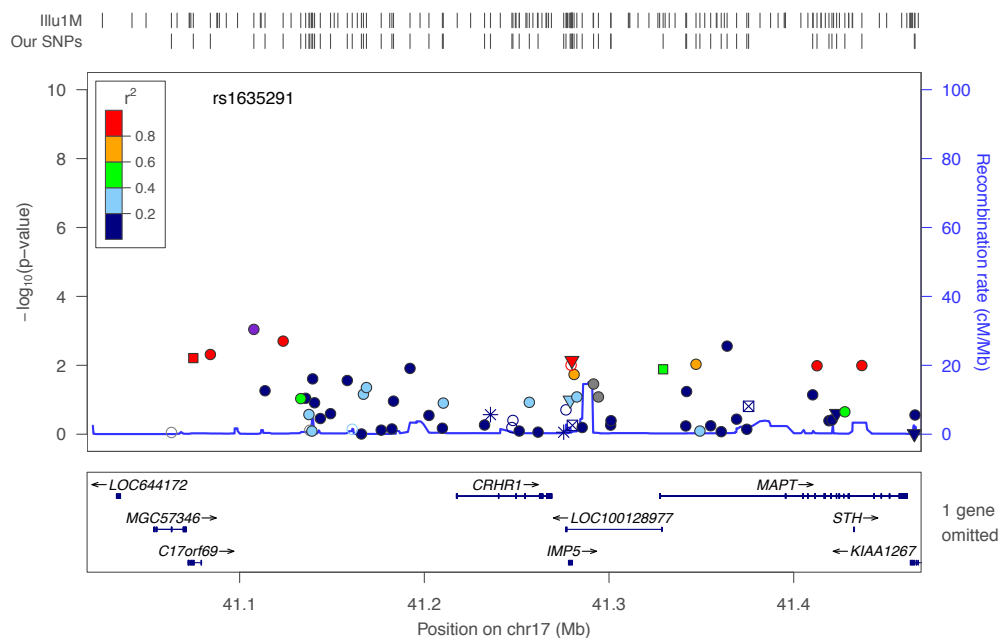

a). *CRHR1*

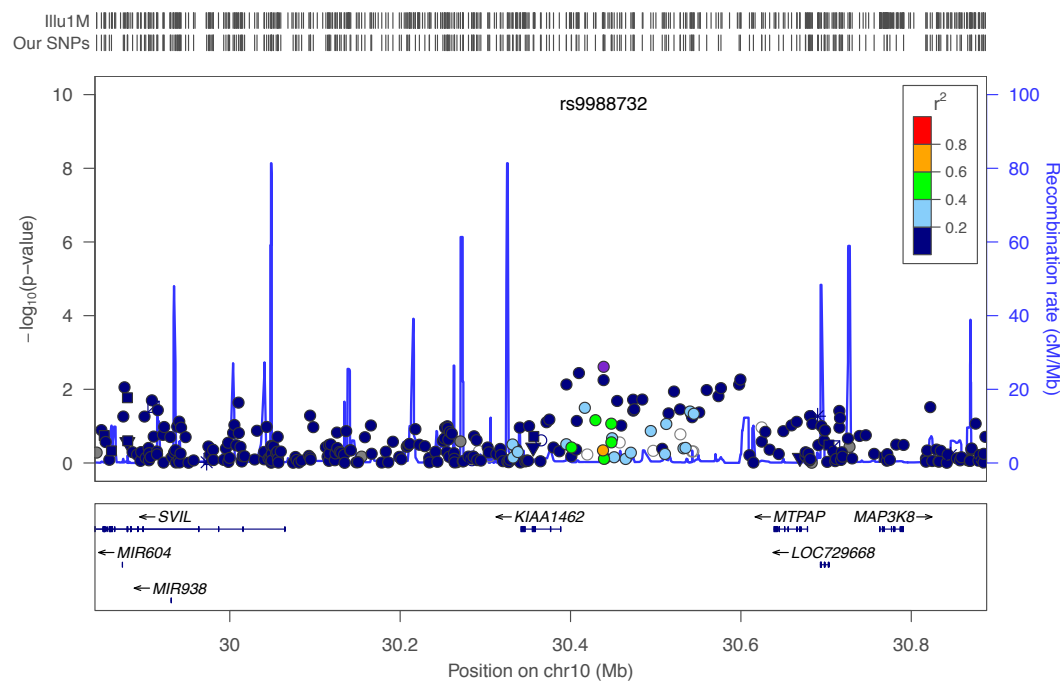

b). *KIAA1462*

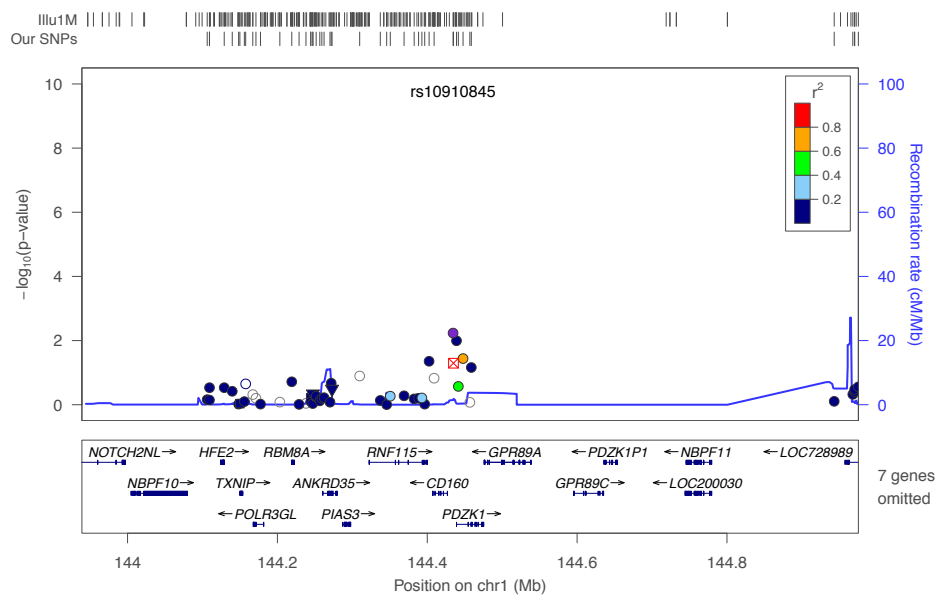

*c).PDZK1*

Supplement: Supplemental Material [file supp_g3.116.035766_FigureS34.pdf]

S35. Locus zoom plot of *SPINK6* in FHS study for NHS\_CNT

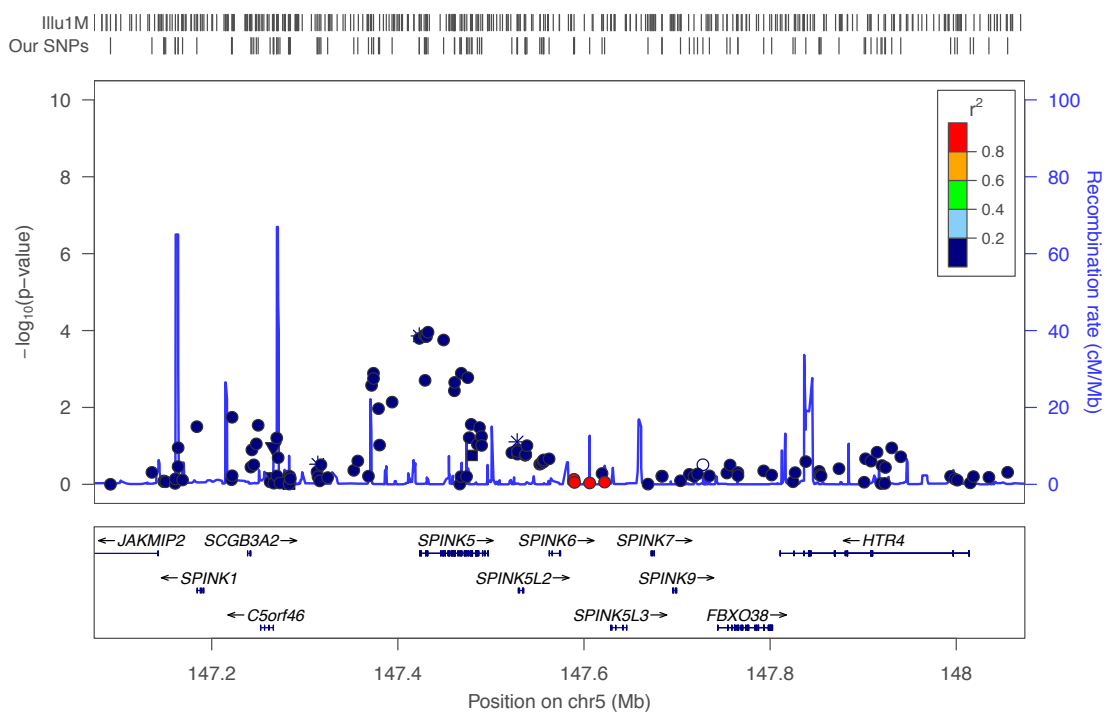

Supplement: Supplemental Material [file supp_g3.116.035766_FigureS35.pdf]

S36. Locus zoom plot of *EVC2* in FHS study for NHS\_CNT

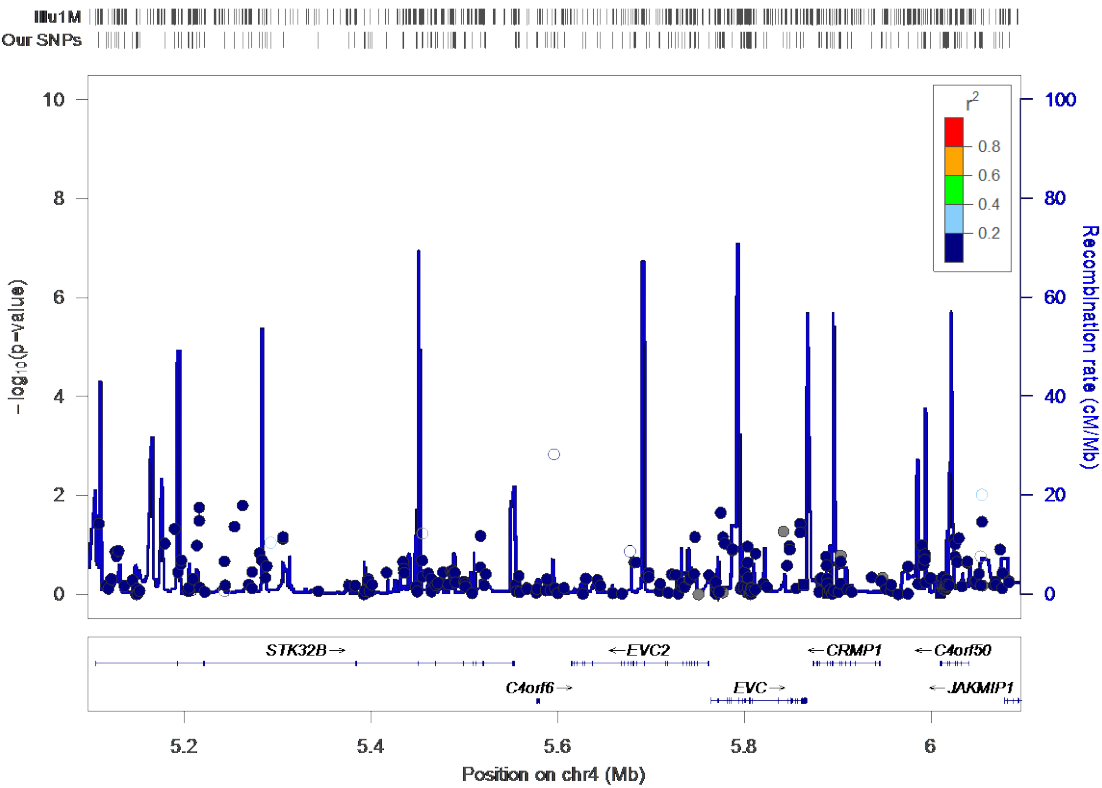

Supplement: Supplemental Material [file supp_g3.116.035766_FigureS36.pdf]
