# Supplementary material for: Genome-Wide Association Study of Meiotic Recombination Phenotypes
Source: G3 (Bethesda). 2016 Oct 12;6(12):3995–4007. doi: 10.1534/g3.116.035766 (PMC5144969; doi:10.1534/g3.116.035766)
Supplement: Supplemental Material [file supp_g3.116.035766_FigureS31.pdf]

S31. Locus zoom plot of Kong et al. top hits.

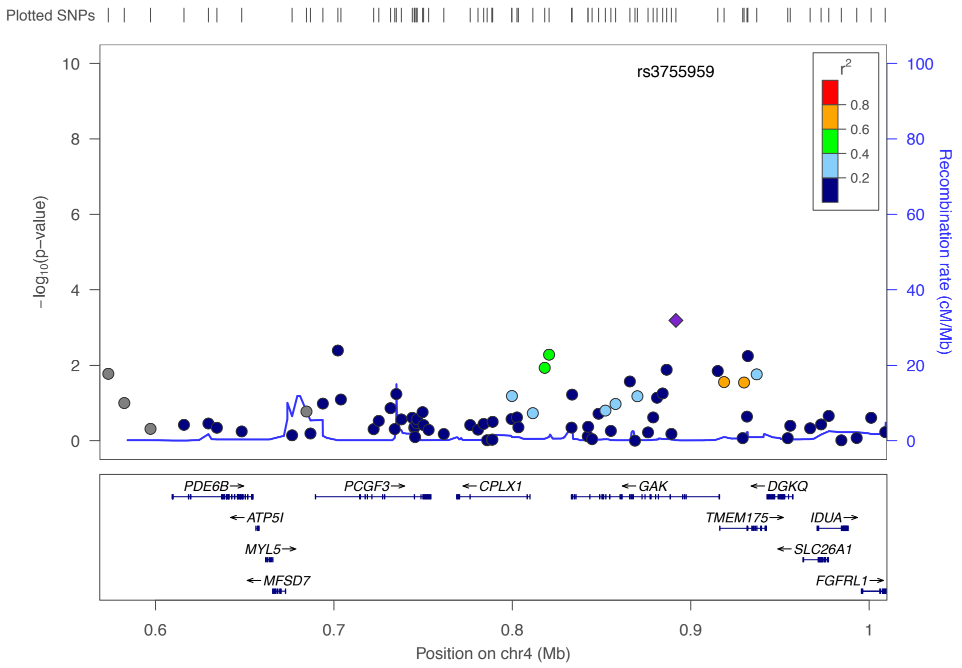

a). HS_PCT(combined); Gene: *CPLX1*

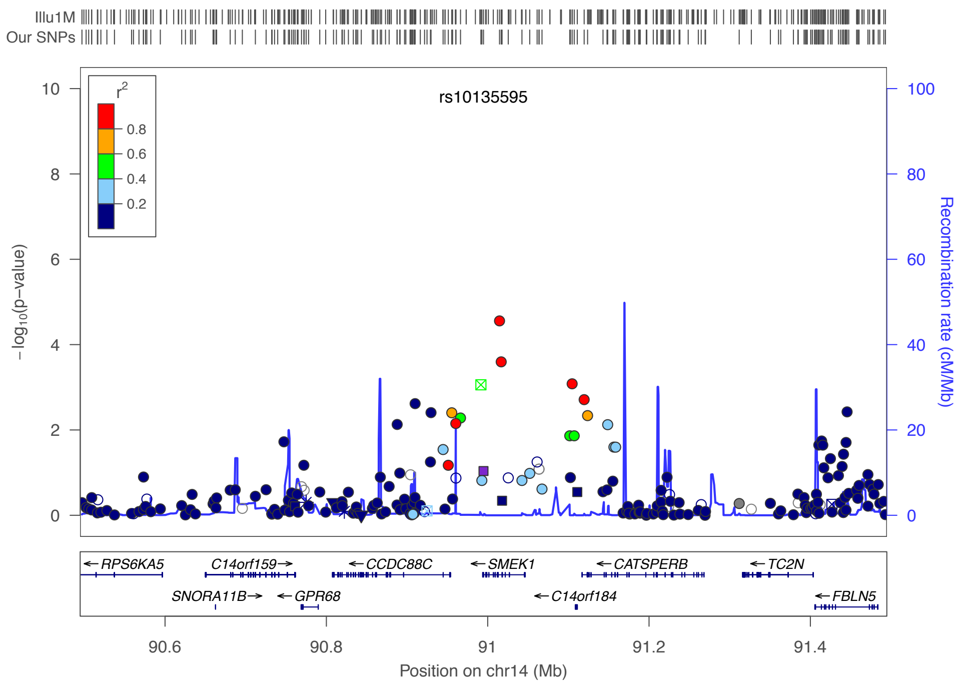

b). ARC (female); Gene: *SMEK1*

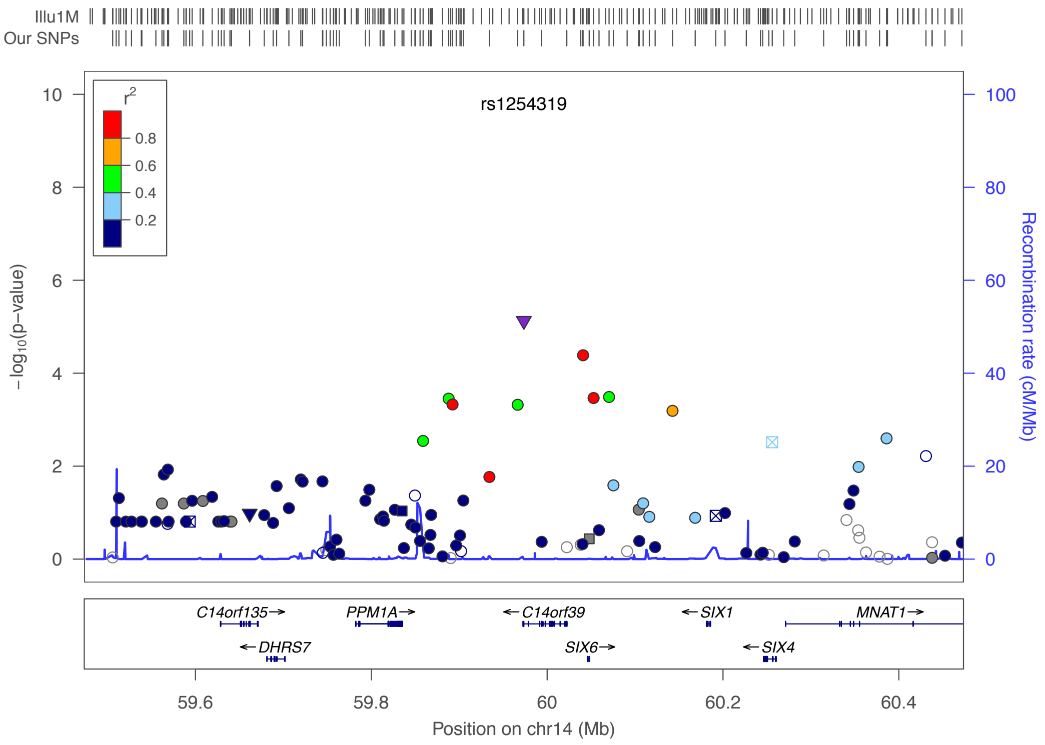

c) ARC(Male), Gene: *C14orf39*
