# Supplementary material for: Genome-Wide Association Study of Meiotic Recombination Phenotypes
Source: G3 (Bethesda). 2016 Oct 12;6(12):3995–4007. doi: 10.1534/g3.116.035766 (PMC5144969; doi:10.1534/g3.116.035766)
Supplement: Supplemental Material [file supp_g3.116.035766_TableS1.pdf]

Table S1: SNPs with lowest *p*-values for MOTIF

Type	SNP	Chr	BP	*p*-value	Direction	Gene list
Combined	*rs4331859*	5	179026713	7.49E-07	----	*RUFY1, HNRNPH1, C5orf60, CBY3, MAML1*
Combined	*rs10872388*	6	132417711	5.58E-06	----	*CTGF, MOXD1*
Combined	*rs1112898*	7	1784522	7.02E-06	++++	*ELFN1, ELFN1, MAD1L1*
Combined	*rs17746897*	4	54930941	8.73E-06	----	*PDGFRA, KIT*
Combined	*rs1854226*	13	97036242	9.01E-06	----	*RAP2A, IPO5*
Female	*rs6886928*	5	166916702	4.38E-06	++	*ODZ2/TENM2, HE578282*
Female	*rs6728479*	2	644471	8.25E-06	++	*TMEM18*
Female	*rs7193684*	16	8046983	8.38E-06	++	*A2BP1*
Female	*rs6055249*	20	7602896	9.11E-06	--	*HAO1*
Female	*rs6487429*	12	24885298	1.09E-05	++	*BCAT1, DAD1L*
Male	*rs1336628*	13	18836533	2.95E-07	+-	*TUBA3C, LOC100101938, TPTE2*
Male	*rs136809*	22	38350821	5.88E-06	--	*CACNA1I*
Male	*rs10882205*	10	95022650	8.11E-06	++	*CYP26C1, CYP26A1, MYOF*
Male	*rs11049351*	12	9234852	1.05E-05	--	*PZP, LOC642846*
Male	*rs11645438*	16	47051221	1.18E-05	--	*LONP2, SIAH1, N4BP1*

Column 6 of the table represents the direction of the effect size of each SNP presented in column 2 in each study. In combined analysis, studies were included in the following order (GDCS female, GDCS male, AGRE female and AGRE male). In female only analysis, first position in the direction column is for GDCS female and the 2nd position is for AGRE female and same ordering is used in male only analysis and for rest of the phenotypes.
