# Supplementary material for: Genome-Wide Association Study of Meiotic Recombination Phenotypes
Source: G3 (Bethesda). 2016 Oct 12;6(12):3995–4007. doi: 10.1534/g3.116.035766 (PMC5144969; doi:10.1534/g3.116.035766)
Supplement: Supplemental Material [file supp_g3.116.035766_TableS2.pdf]

Table S2: Replication of Kong et al. (2014) findings

**Chromosome Gene**	**Association in Icelandic population (all associations with ARC except as noted)**	**Replication of Kong's significant hits in our analyses**
Chr: 4<br>Gene: *CPLX1*	Primarily associated in females. Very rare variant.	In females, a SNP in the nearby *GAK* gene gave $p<10^{-5}$. The FHS data set showed a signal ($p\sim10^{-7}$) between *CPLX1* and *GAK*. See Figure 6.
Chr: 14<br>Gene: *CCNB1IP1*	Primarily associated in females	This region had poor coverage in the GDCS and AGRE data sets, but we observed $p<10^{-3}$ for nearby SNPs in females. In FHS, males show $p\sim10^{-3}$.
Chr: 14<br>Gene: *C14orf39*	Female only.	Strong hit (for our sample size) in males (10^{-6}) for ARC. Suggestion of replication in females ($p\sim10^{-3}$). Suggestive association with NHS_CNT in males in nearby gene ($p\sim10^{-5}$). FHS shows replication in males for HS_PCT ($p\sim10^{-4}$).
Chr: 14<br>Gene: *SMEK1*	Female only	Replicated in females (10^{-5}). FHS also shows hint of replication in females for ARC, and for HS_PCT ($p\sim10^{-4}$) in combined analysis.
Chr: 20<br>Gene: *RAD21L*	Primarily associated in males	This region had poor coverage in the GDCS and AGRE data sets. In FHS males, no hint of replication.
Chr: 1<br>Gene: *MSH4*	Female only. Very rare variant. Hint of hotspot effect.	Good coverage in GDCS and AGRE data sets. This variant is very rare in our study population (MAF: .009) and this particular variant is not present in our chip. Hint of replication ($p\sim10^{-3}$) among males for MOTIF phenotype.
Chr: 17<br>*CCDC43*	Primarily associated in males	This region had poor coverage in the GDCS and AGRE data sets. The nearest SNP shows p-value 10^{-3} in males for HS_PCT and HS_CNT. Female also shows similar p-value for ARC and HS_CNT.
