# Supplementary material for: Genome-Wide Association Study of Meiotic Recombination Phenotypes
Source: G3 (Bethesda). 2016 Oct 12;6(12):3995–4007. doi: 10.1534/g3.116.035766 (PMC5144969; doi:10.1534/g3.116.035766)
Supplement: Supplemental Material [file supp_g3.116.035766_TableS3.pdf]

Table S3: SNPs with lowest *p*-values for phenotype ARC in FHS

**Analysis Type**	**SNP**	**Chr**	**BP**	***p*-value**	**Gene list**
Combined	*rs2282127*	20	3740203	1.625e-10	*HSPA12B, SPEF1, CENPB, MAVS*
Combined	*rs12040859*	1	22024780	3.625e-09	*USP48, LDLRAD2, HSPG2*
Combined	*rs4765556*	12	123421228	5.069e-08	*FAM101A, NCOR2*
Combined	*rs9288898*	3	96586929	8.099e-08	*LOC255025*
Combined	*rs9982042*	21	21833266	1.357e-07	*NCAM2*
Female	*rs4751430*	10	129186708	2.046e-07	*DOCK1, NPS, FAM196A*
Female	*rs17152325*	7	105358723	1.213e-06	*ATXN7L1, CDHR3*
Female	*rs6596770*	5	107540043	3.545e-06	*FBXL17*
Female	*rs7732894*	5	120964498	8.071e-06	*FTMT*
Female	*rs1571317*	13	36382692	1.058e-05	*SMAD9,ALGS, RFXAP*
Male	*rs2282127*	20	3740203	2.312e-11	*HSPA12B, SPEF1, CENPB, MAVS*
Male	*rs12040859*	1	22024780	3.323e-09	*USP48, LDLRAD2, HSPG2*
Male	*rs2878441*	4	827272	2.777e-08	*CPLX1, GAK*
Male	*rs16917322*	8	96176568	2.815e-08	*C8orf38, PLEKHF2*
Male	*rs4765556*	12	123421228	4.799e-08	*FAM101A, NCOR2*
